# Critical assessment of sequence-based protein-protein interaction prediction methods that do not require homologous protein sequences

**DOI:** 10.1186/1471-2105-10-419

**Published:** 2009-12-14

**Authors:** Yungki Park

**Affiliations:** 1 Institute of Cellular and Molecular Biology (MBB 3 210B), Center for Systems and Synthetic Biology, University of Texas at Austin, 2500 Speedway, Austin, Texas, USA

## Abstract

**Background:**

Protein-protein interactions underlie many important biological processes. Computational prediction methods can nicely complement experimental approaches for identifying protein-protein interactions. Recently, a unique category of sequence-based prediction methods has been put forward - unique in the sense that it does not require homologous protein sequences. This enables it to be universally applicable to all protein sequences unlike many of previous sequence-based prediction methods. If effective as claimed, these new sequence-based, universally applicable prediction methods would have far-reaching utilities in many areas of biology research.

**Results:**

Upon close survey, I realized that many of these new methods were ill-tested. In addition, newer methods were often published without performance comparison with previous ones. Thus, it is not clear how good they are and whether there are significant performance differences among them. In this study, I have implemented and thoroughly tested 4 different methods on large-scale, non-redundant data sets. It reveals several important points. First, significant performance differences are noted among different methods. Second, data sets typically used for training prediction methods appear significantly biased, limiting the general applicability of prediction methods trained with them. Third, there is still ample room for further developments. In addition, my analysis illustrates the importance of complementary performance measures coupled with right-sized data sets for meaningful benchmark tests.

**Conclusions:**

The current study reveals the potentials and limits of the new category of sequence-based protein-protein interaction prediction methods, which in turn provides a firm ground for future endeavours in this important area of contemporary bioinformatics.

## Background

Protein-protein interaction (PPI) plays a central role in many biological processes. Information on PPIs can hint at potential functions for uncharacterized proteins [1]. On a broader scale, PPI networks allow for a systems-level understanding of molecular processes underpinning life [2]. Powered by high-throughput techniques, yeast two-hybrid screens have been applied on a genomic scale to several organisms for a systematic identification of PPIs [3-9]. Related techniques have also been developed, allowing researchers to address different aspects of PPIs than yeast two-hybrid screens [10,11]. On the other hand, PPIs in protein complexes have been investigated by affinity purification followed by mass spectrometry analysis [12,13].

Concurrently, there have been intensive efforts to develop computational methods for predicting PPIs. Early approaches tried to mine patterns from genomic data that are a priori expected for PPIs such as gene neighborhoods and gene order [14], the existence of fusion genes [15,16], the co-evolution of interaction partners [17], phylogenetic profiles [18] and similarity of phylogenetic trees [19,20]. Some of these ideas have recently been explored again in a refined manner [21,22]. Since domain-domain interactions underlie many PPIs, they have also been intensively studied [23-37]. More generalized concepts than protein domains, such as linear sequence motifs or sets of discontinuous sequence motifs defined on the basis of protein structures, have also been explored [38-48]. Approaches combining different types of data in a self-consistent manner have been put forward [49,50]. In addition, microarray gene expression data have been explored as a potential source for predicting PPIs [51-53].

Recently, a unique category of sequence-based prediction methods has been put forward - unique in the sense that it does not require homologous protein sequences [54-58]. This enables it to be universally applicable to all protein sequences unlike many of previous sequence-based prediction methods. For example, domain-based methods do not work for query protein pairs without domain information, and the Rosetta-stone methods [15,16] and the co-evolution-based methods [17-21] can not be applied to proteins without homologous protein sequences. The new sequence-based, universally applicable prediction methods would have far-reaching utilities in many fields of biology research, if effective as claimed. Upon close survey, however, I realized that many of them were not properly benchmarked, e.g., tested on ill-sized data sets often fraught with homologous proteins. Moreover, newer methods were often published without performance comparison with previously proposed ones. Thus, it is not clear how good they are and whether there are significant performance differences among them. These are important issues to investigate for both a true advancement of this research field and maximizing the benefits of computational predictions for the general research community. In this work, I have implemented and thoroughly tested four different methods using large-scale, non-redundant data sets to address these issues.

## Results and Discussion

### Four methods for comparative benchmarking

In this study, I tested 4 different methods. The selection criteria were 1) the original purpose of the method was to predict physical binary PPIs, 2) the method is sequence-based, yet does not require homologous protein sequences and 3) either trainable versions of the software are available or the description in the original report is specific enough for me to confidently implement it on my own. The four methods are as follows.

• M1: the signature product-based method proposed by Martin and co-workers [55]. In this method, the sequence information for a protein pair is encoded by a product of signatures, which is then classified by a support vector classifier (SVC) [59]. For individual proteins, signatures are defined to be a culled set of subsequences. I used their "sym" kernel since preliminary analysis showed that it worked slightly better than their Gaussian kernel, i.e., exp(-0.5× [sym((A, B),(A, B)) - 2×sym((A, B),(C, D)) + sym((C, D),(C, D))]), where sym((A, B),(C, D)) is the "sym" kernel for a pair of protein pairs A-B and C-D, and sym((A, B),(A, B)) and sym((C, D),(C, D)) are analogously defined. For the details, please refer to the original paper [55].

• M2: the method developed by Pitre and coworkers, also known as PIPE [58,60]. For a pair of proteins, PIPE looks for the co-occurrences of their subsequences in protein pairs that are known to interact.

• M3: the method introduced by Shen and coworkers [57]. In this method, a protein sequence is represented by a reduced set of amino acids. Then, each protein sequence is encoded by a feature vector that represents the frequencies of 3 amino acid-long subsequences. The feature vectors are then concatenated for a pair of proteins and classified by an SVC.

• M4: the method developed by Guo and coworkers [56]. A feature vector for a protein sequence comprises its auto-correlation values of 7 different physicochemical scales. The feature vectors are then concatenated for a protein pair and classified by an SVC.

### Cross-validation on the yeast and the human data

**Figure 1:**
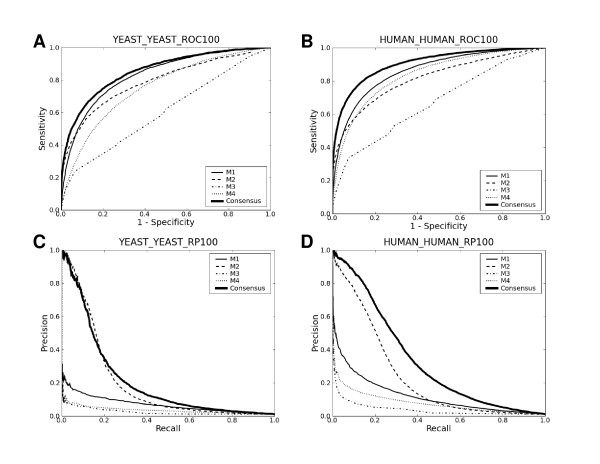
**Cross-validation on the yeast and the human data**. ROC and recall-precision plots for the four tested methods and the consensus method. The title of each plot is of the 'D1_D2_(ROC|RP)100' format, where D1 is the training data set, D2 is the testing data set, and '100' indicates that the size of the negative test data set is 100 times that of the positive test data set. 'ROC' indicates an ROC plot whereas 'RP' indicates a recall-precision plot. When D1 and D2 are identical as is here, 4-fold cross-validation was performed. When not identical, D1 and D2 are used for training and testing, respectively.

I first estimated their performance on the yeast and the human data in 4-fold cross-validation (Fig. 1 and Table 1). The following points are apparent in Table 1. First, M1 significantly excels the others in terms of the area under the receiver-operating characteristic (ROC) curve (AUC) across both the yeast and the human data: see the Additional File 1 for detailed *p *values. Second, M2 significantly outperforms the others in terms of recall-precision across both the yeast and the human data. Third, M3 is least effective regardless of which performance measure to use for comparison. The dominance order among the four methods is the same for both the yeast and the human data, in spite of the fact that each data set is uniquely biased (see below). Moreover, these three points are repeatedly observed in other analyses presented below. Thus, the analysis in Fig. 1 and Table 1 appears to unravel genuine performance differences.

**Table 1:** Cross-validation results on the yeast and the human data

	Yeast 10*N*^1^		Yeast 100*N*	
	AUC	P20R^2^	AUC	P20R
M1	0.83	0.55	0.83	0.11
M2	0.79	0.82	0.79	0.33
M3	0.60	0.28	0.60	0.04
M4	0.75	0.35	0.75	0.05
C	0.85	0.84	0.85	0.34

	**Human 10*N*^1^**		**Human 100*N***	
	**AUC**	**P20R**	**AUC**	**P20R**

M1	0.86	0.70	0.86	0.19
M2	0.81	0.91	0.81	0.51
M3	0.67	0.36	0.67	0.05
M4	0.83	0.59	0.83	0.12
C	0.91	0.95	0.90	0.67

^1^Evaluation using a negative subset of size 10*N *randomly chosen from the 100*N *negative set, where *N *is the size of the positive set.

^2^Precision values at 20% recall

Another point worth discussing is the use of two complementary performance measures in the above analysis. AUCs are a widely used figure for assessing the performance of computational prediction methods. Since AUCs are solely based on ranks of positives relative to those of negatives, AUCs are to a large extent insensitive to absolute numbers of false positives. This may be a significant drawback. For example, for experimental biologists who want to use prediction results for prioritizing candidates for in-depth experimental follow-ups, the absolute number of false positives may equally matter. Thus, estimation of prediction performance by AUCs alone can be misleading if absolute numbers of false positives become as relevant. In this regard, recall-precision analysis is complementary to AUCs because it is sensitive to absolute numbers of false positives. For a clear demonstration of this point, prediction performance for each method was re-estimated using the original positive set (size *N*) and a negative subset of size 10*N *randomly chosen from the original 100*N *negative set. By reducing the negative set size from 100*N *to 10*N*, we effectively reduced the number of potential false positives by 10 fold. As shown in Table 1, AUCs change little between the 10*N *and the 100*N *sets for all four methods. Yet, the P20R values (precision at 20% recall; see the Methods section) dramatically improve for all four methods for the 10*N *set compared to the 100*N *set. Similar improvements are also obvious in recall-precision plots (not shown). Improvements coming from the use of the 10*N *set instead of the 100*N *set are, of course, not real: they are just artefacts coming from the use of ill-sized negative data. The number of potential protein-protein pairings is expected to be > 100 times the number of PPIs in the cell. In this sense, negative sets of size 10*N *are grossly ill-sized, let alone 1*N *sets that were used for benchmarking for some of the four methods in the original reports. Even the 100*N *set may not still be large enough. However, prohibitively high computational expenses made it very difficult to use significantly larger ones. Taken together, Table 1 illustrates the importance of complementary performance measures along with data sets right-sized in a physiological sense for meaningful performance estimation of prediction methods. Given the importance of right-sized negative data sets for meaningful benchmark tests, all the results reported hereafter are based on negative data sets of size 100*N*, unless otherwise stated.

As noted above, M1 dominates in terms of AUC while M2 excels in terms of recall-precision. This dominance reversal between AUC and recall-precision may be inferred by the cross between the ROC plot of M1 and that of M2 in Fig. 1. M2 is based on counting how frequently pairs of subsequences in the query protein pair occur in protein pairs known to interact. When the count is low, its prediction outcome is no interaction. Since the count is based on similarity of 20 amino acid-long segments, it is more often low than high. This conservative prediction behavior is thought to underlie its good performance in terms of recall-precision. This core idea of M2 has been successfully exploited in one form or another by other related prediction methods [38-43].

The performance of M1 reported here appears not to be as good as that reported in Fig. 1 of the original paper [55]. A very likely reason for this is that the two studies adopt different definitions for true positives: the current study defines a true positive as a pair of proteins known to interact and predicted to interact, whereas the source code for M1 that I downloaded from the original authors' website defines a true positive as a pair of proteins assumed not to interact and predicted not to interact.

### Insight into the performance difference between M1 and M3

The performance contrast between M1 and M3 is interesting, given that their approaches stem from overall similar ideas. Methodological differences between them can be decomposed into 1) feature representation of individual proteins and 2) how to combine the features of individual proteins to represent protein pairs. We investigated the effects of the second factor on prediction performance because this is a recurring issue whenever it is necessary to encode protein pairs rather than individual proteins. M1 computes the outer product of individual feature vectors (i.e., **ab**^T ^for two column vectors **a **and **b**) while M3 concatenates them. Specifically, we wanted to investigate which of the two approaches - computing the outer product of individual feature vectors as in M1 and concatenating them as in M3 - leads to better prediction performance. To this end, we modified M1 such that the outer product of individual feature vectors is replaced by their concatenation, and the modified M1 was tested on the yeast and the human data as in Table 1. Table 2 summarizes the results. A comparison of M1's performance in Tables 1 and 2 indicates that the two approaches lead to similar prediction performance, even though the performance of M1 in Table 1 is significantly better than that of M1 in Table 2 (all four *p *values < 10^-7^). This suggests that the outer product approach for encoding protein pairs is not a critical factor for the success of M1. Conversely, this suggests that the poor performance of M3 is mostly attributable to its less effective feature representation of individual proteins. At first glance, this may seem odd because the feature encoding system of M1 may look similar to that of M3. The feature encoding system of M1 is, however, much more sophisticated than that of M3. M1's feature vectors are culled sets of 3 amino acid-long subsequences that are based on 20 naturally occurring amino acid types whereas M3's feature vectors are full sets of 3 amino acid-long subsequences that are based on a reduced set of 7 "amino acid" types. For efficient handling of large feature vectors, M1 was implemented using special data structures [55]. Apparently, these seemingly small differences led to considerable performance differences. In this regard, it is also to be noted that a previous study has shown that feature vector encoding systems like that of M3 do not work well for PPI predictions [45]. The respectable performance of M4 suggests that protein pairs that interact do display some physicochemical properties that not all potential protein pairs share.

**Table 2:** Prediction performance of the modified M1 on the yeast and the human data

	Yeast 100*N*		Human 100*N*	
	AUC	P20R	AUC	P20R
M1	0.82	0.10	0.84	0.17

### Cross-species benchmarking

In the above analyses, prediction methods were trained and tested on the same species data in 4-fold cross-validation. What about training prediction methods on the yeast data and testing them on the human data or vice versa? This is a relevant question to ask because many prediction servers have been trained on one species' data and yet predict also for other species' protein pairs. Although it is not fully clear whether PPIs taking place in yeast are of a fundamentally different nature than those taking place in human, yeast PPI data that are typically used for training prediction methods are certainly expected to contain distinct biases from human PPI data. As such, it is not clear, for example, whether prediction methods trained with the human data work as well on the yeast data as when trained with the yeast data.

**Figure 2:**
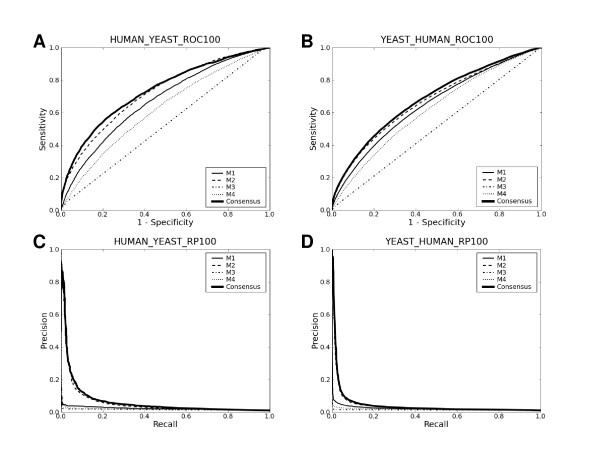
**Cross-species benchmarking results**. The title of each plot reads in the same way as in Fig. 1.

In Table 3 and Fig. 2, we trained prediction methods with the human data and tested them on the yeast data and vice versa. Comparison of Table 3 with Table 1 reveals that prediction methods are much more effective on the yeast data when trained with the yeast data than when trained with the human data. This is in spite of the fact that much more data points were used during training with the human data (34862 data points) than during training with the yeast data (~5800 data points in 4-fold cross-validation). This strongly suggests that data sets typically used for training prediction methods contain peculiar biases that limit the general applicability of prediction methods trained with them. Likewise, prediction methods are more effective on the human data when trained with the human data than when trained with the yeast data. However, in this case, the asymmetric numbers of data points used for the two trainings might also have affected the results. In sum, this analysis indicates that prediction methods trained only with particular PPI data sets are likely to have greater generalization errors than those suggested by cross-validation with such particular sets - a point overlooked by many of the four methods in their original benchmarks.

**Table 3:** Cross-species testing results

	Human - Yeast^1 ^100*N*		Yeast - Human 100*N*	
	AUC	P20R	AUC	P20R
M1	0.67	0.03	0.65	0.03
M2	0.72	0.06	0.67	0.04
M3	0.52	0.02	0.51	0.01
M4	0.62	0.02	0.62	0.02
C	0.73	0.07	0.68	0.04

^1^"A - B" signifies training with the A data and testing on the B data.

In this Table, prediction methods were trained with all the data from one species and tested on all the data from another species (no 4-fold cross-validation).

### Combined set benchmarking

**Figure 3:**
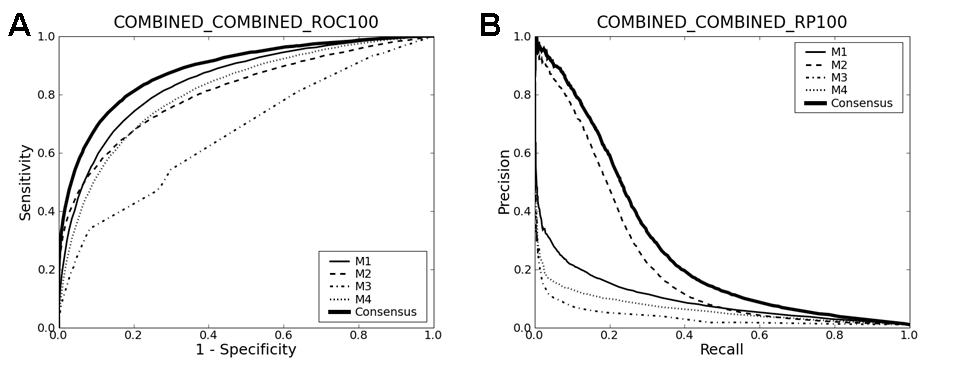
**Cross-validation on the combined data**. The title of each plot reads in the same way as in Fig. 1.

The above analysis suggests that one straightforward way of developing generally applicable prediction methods is to use diverse training data so that they learn only features common to diverse data. To test this idea, I trained the four methods on the data that combines the yeast and the human data (the combined set). Then, their prediction performance was evaluated for three different sets (the yeast data, the human data and the combined data) in 4-fold cross-validation. Fig. 3 and Table 4 summarize the results. First, the inclusion of the yeast data did not significantly affect the prediction performance of all four methods on the human data. This may be due to the fact that the size of the human data is ~4.5 times larger than that of the yeast data, dominating the combined set. Second, the inclusion of the human data slightly degraded the performance of some methods (M1 and M2) on the yeast data, although the results in Table 4 are much better than those in Table 3.

**Table 4:** Testing results on the combined data set

	Combined - Yeast 100*N*		Combined - Human 100*N*		Combined - Combined 100*N*	
	AUC	P20R	AUC	P20R	AUC	P20R
M1	0.79	0.07	0.86	0.18	0.85	0.15
M2	0.79	0.24	0.82	0.52	0.81	0.48
M3	0.62	0.04	0.68	0.05	0.67	0.05
M4	0.74	0.05	0.83	0.11	0.81	0.10
C	0.84	0.31	0.89	0.63	0.88	0.59

### Consensus approach

Having carried out a thorough comparative analysis for the four methods, a naturally arising question is how good their performance is. Another formulation of this question would be "would it be easy to develop another method that consistently outperforms the four methods in terms of both AUC and recall-precision?" Since the primary interest in this work is not to develop a novel method that surpasses existing ones, I touched on this issue simply by designing a consensus approach and asking how it compares with the four methods. As described in the Methods section, I tried an SVC with a linear kernel as a simple consensus approach, with all its parameters set to default values. In this case, the feature vector consisted of classification scores generated by the four methods.

Tables 1, 3 and 4 and Figs. 1, 2 and 3 summarize the results ("C" below M4 in each Table). The consensus approach consistently outperforms all four methods in terms of both AUC and recall-precision, and this is even without any serious attempts to optimize SVC parameters. These results strongly suggest that there is still ample room for further developments. The use of the linear kernel in the consensus methods makes it possible to look into how much each method contributes to them. Table 5 lists the mean coefficients of each method for each data set. The mean coefficients were normalized by dividing by their sum. The large contributions of M1 and M2 to the consensus methods are consistent with the results presented above, as is the least contribution of M3. However, since the four methods are not "orthogonal" to each other, other drastically different linear combinations of the four methods could lead to separating hyperplanes as optimal as the one with the coefficients in Table 5. In other words, the redundancy of the four methods makes it difficult to infer, from the magnitudes of the linear SVC coefficients, how useful each method is in forming the consensus methods.

**Table 5:** Mean coefficients of the four methods in the linear SVC consensus methods

	Yeast	Human	Combined
M1	0.35	0.30	0.13

M2	0.42	0.26	0.52

M3	0.08	0.12	0.11

M4	0.15	0.32	0.24

One potential way of evaluating the usefulness of the four methods while overcoming the redundancy is to form consensus methods that selectively include component methods (M1 through M4) and compare their performance with that of full consensus methods that incorporate all four. Table 6 shows the results for all possible combinations of 2 or 3 methods, revealing the following points. First, methods that combine M1 and M4 favourably rival full consensus methods in terms of both AUC and recall-precision. This is rather surprising because M1 and M4 tended to be much worse than M2 in terms of recall-precision in the above analyses. For this reason, it was expected that consensus methods should incorporate M2 in order to be good in terms of recall-precision. Apparently, the simple linear SVC could learn how to combine M1 and M4 in such a way that the combined predictions are now good not only in terms of AUC but also in terms of recall-precision, even without M2. Second, as expected, it is consistently observed that exclusion of M1 leads to decrease in AUC values. This is also true for M4 to some extent. Third, the presence of M2 does not necessarily lead to good performance in terms of recall-precision. In sum, M1 and M4 appear to be sufficient to fully account for the success of the full consensus methods.

**Table 6:** Prediction performance of consensus methods that combine two or three methods

Results on the yeast data											
	**Full model**	**M2-M3-M4**	**M1-M3-M4**	**M1-M2-M4**	**M1-M2-M3**	**M1-M2**	**M1-M3**	**M1-M4**	**M2-M3**	**M2-M4**	**M3-M4**

AUC	0.85	0.81	0.85	0.85	0.85	0.85	0.85	0.85	0.79	0.81	0.81

P20R	0.34	0.37	0.34	0.35	0.29	0.29	0.29	0.35	0.32	0.38	0.37

**Results on the human data**											

	**Full model**	**M2-M3-M4**	**M1-M3-M4**	**M1-M2-M4**	**M1-M2-M3**	**M1-M2**	**M1-M3**	**M1-M4**	**M2-M3**	**M2-M4**	**M3-M4**

AUC	0.90	0.88	0.90	0.90	0.89	0.89	0.89	0.90	0.82	0.88	0.88

P20R	0.67	0.63	0.67	0.67	0.64	0.64	0.64	0.67	0.52	0.62	0.63

**Results on the combined data**											

	**Full model**	**M2-M3-M4**	**M1-M3-M4**	**M1-M2-M4**	**M1-M2-M3**	**M1-M2**	**M1-M3**	**M1-M4**	**M2-M3**	**M2-M4**	**M3-M4**

AUC	0.88	0.86	0.88	0.88	0.86	0.86	0.86	0.88	0.81	0.86	0.86

P20R	0.59	0.54	0.59	0.59	0.54	0.54	0.54	0.59	0.48	0.54	0.54

**Results on the cross-species testing, Human - Yeast^1^**											

	**Full model**	**M2-M3-M4**	**M1-M3-M4**	**M1-M2-M4**	**M1-M2-M3**	**M1-M2**	**M1-M3**	**M1-M4**	**M2-M3**	**M2-M4**	**M3-M4**

AUC	0.73	0.71	0.73	0.73	0.73	0.73	0.73	0.73	0.72	0.71	0.71

P20R	0.07	0.07	0.07	0.07	0.06	0.06	0.06	0.07	0.06	0.07	0.07

**Results on the cross-species testing, Yeast - Human^1^**											

	**Full model**	**M2-M3-M4**	**M1-M3-M4**	**M1-M2-M4**	**M1-M2-M3**	**M1-M2**	**M1-M3**	**M1-M4**	**M2-M3**	**M2-M4**	**M3-M4**

AUC	0.68	0.65	0.68	0.68	0.67	0.67	0.67	0.68	0.67	0.65	0.65

P20R	0.04	0.03	0.04	0.04	0.03	0.03	0.03	0.04	0.04	0.03	0.03

^1^"A - B" signifies training with the A data and testing on the B data.

### Analysis of prediction results by protein types

Would there be any protein types that could be better predicted by the prediction methods tested in this study? Could it be that some methods significantly outperform others for special categories of proteins, even though their overall performance as shown above is not as good as that of others? To address these issues in a systematic way, I analyzed the prediction results by the gene ontology (GO) slims [61]. The GO slim annotations for the yeast and the human proteins were downloaded from the GO project website. Altogether 128 GO terms were considered. For each combination of a data set (the yeast, the human or the combined data) and an evaluation scheme (AUC or P20R), Table 7 lists five GO terms for which best performance was achieved. The complete results are available in Additional File 2. A first obvious point in Table 7 is that the consensus method is the best-performing method in terms of AUC. In terms of P20R, it is either the consensus method or M2 that is most effective. This effectiveness of M2 in terms of P20R is consistent with the analysis shown above. Another obvious point in Table 7 is that the GO terms for which best performance was achieved in the yeast cross-validation do not overlap those for which best performance was achieved in the human cross-validation. This appears to reflect the distinct biases in the yeast and the human data sets, as shown above in the cross-species benchmark tests. Finally, GO terms for which good performance was achieved in terms of AUC tend to overlap those for which good performance was achieved in terms of P20R. Specifically, the Spearman's rank correlation coefficients between the ranking according to AUC and that according to P20R are 0.67 (*p *value < 2.2×10^-16^), 0.68 (*p *value < 5.2×10^-10^) and 0.77 (*p *value < 2.2×10^-16^) for the yeast, the human and the combined data, respectively. This indicates that a selective use of prediction methods for proteins with such GO terms may yield more fruitful results. It is to be noted that the prerequisite for this is just either protein having such GO annotations because the analysis in Table 7 was based on GO terms applying to either protein in protein pairs.

**Table 7:** Analysis of prediction results by the gene ontology slims

Results on the yeast data sorted according to AUC					
	**GO term**	**# of cases**	**Best method**	**AUC**	**GO term explanation**

1	0005198	39513	C^1^	0.90	Structural molecular activity

2	0007124	9192	C	0.89	Pseudohyphal growth

3	0006997	10093	C	0.89	Nucleus organization

4	0007047	18668	C	0.89	Cell wall organization

5	0005215	44019	C	0.89	Transporter activity

**Results on the yeast data sorted according to P20R**					

	**GO term**	**# of cases**	**Best method**	**P20R**	**GO term explanation**

1	0005618	8689	M2	1.00	Cell wall

2	0006997	10093	C	0.97	Nucleus organization

3	0042254	44304	C	0.95	Ribosome biogenesis

4	0005198	39513	C	0.92	Structural molecule activity

5	0008289	10690	M2	0.92	Lipid binding

**Results on the human data sorted according to AUC**					

	**GO term**	**# of cases**	**Best method**	**AUC**	**GO term explanation**

1	0008907	245	C	1.00	Integrase activity

2	0004871	71939	C	0.92	Signal transducer activity

3	0051704	88280	C	0.92	Multi-organism process

4	0008219	98990	C	0.92	Cell death

5	0016740	244001	C	0.91	Transferase activity

**Results on the human data sorted according to P20R**					

	**GO term**	**# of cases**	**Best method**	**P20R**	**GO term explanation**

1	0009405	1017	M2	1.00	Pathogenesis

2	0008907	245	M2	1.00	Integrase activity

3	0004871	71939	C	0.91	Signal transducer activity

4	0004872	208752	C	0.88	Receptor activity

5	0016301	110554	C	0.88	Kinase activity

**Results on the combined data sorted according to AUC**					

	**GO term**	**# of cases**	**Best method**	**AUC**	**GO term explanation**

1	0008907	245	C	0.99	Integrase activity

2	0004871	77553	C	0.92	Signal transducer activity

3	0015267	7183	C	0.91	Channel activity

4	0004872	208752	C	0.91	Receptor activity

5	0051704	88280	C	0.91	Multi-organism process

**Results on the combined data sorted according to P20R**					

	**GO term**	**# of cases**	**Best method**	**P20R**	**GO term explanation**

1	0005618	8689	M2	1.00	Cell wall

2	0009405	1017	M2	1.00	Pathogenesis

3	0008907	245	M2	1.00	Integrase activity

4	0006997	10093	M2	0.97	Nucleus organization

5	0008289	10690	M2	0.92	Lipid binding

^1^C: the consensus method that integrates the four methods M1 through M4.

For each combination of a data set (the yeast, the human or the combined data) and an evaluation scheme (AUC or P20R), five GO terms are listed for which best performance was achieved. For each GO term, the number of protein-protein pairs in the data set is shown in the third column for which either protein in the pair is annotated with that GO term. Also shown are the best-performing method (column 4) and its performance (column 5).

## Conclusions

In this work, I have implemented and thoroughly tested four different sequence-based PPI prediction methods that do not require homologous protein sequence. It revealed 1) significant performance differences among them and 2) ample room for further developments. In addition, it illustrated the importance of complementary performance measures along with right-sized data sets for meaningful benchmark tests. Thus, the current work provides a firm ground for future endeavors in computational prediction of protein-protein interactions. Regarding practical use of predicted PPIs in experimental biological research, PPI prediction results may be best used in conjunction with other types of biological data.

## Methods

### Data sources

Yeast PPI data were collected from the *Saccharomyces cerevisiae *core subset of the Database of Interacting Proteins (DIP) [62]. Human PPI data were collected from the Human Protein Reference Database [63]. The PPI data from the two databases were refined as follows. First, for each species (yeast and human), a representative set of non-redundant protein sequences at the identity level of 40% was generated by clustering analysis with the CD-HIT program [64]. Second, proteins whose length is less than 50 amino acids were removed. This refinement process led to 3867 and 17431 positive interactions for yeast and human, respectively. High-quality negative PPI data (i.e. protein pairs that are known not to interact) are also needed for benchmarking, yet are not readily available. Thus, one has to make up one on the basis of a priori assumptions (e.g., proteins that reside in different subcellular locations tend not to interact). A thorough analysis by Ben-Hur and Noble [65] showed that one of the best ways of generating negative PPI data is to randomly pair proteins that are not known to interact. Thus, I generated negative PPI data for each species by randomly pairing proteins from its positive set. I made sure that no such random pairs appear in the respective positive set. All the data sets used in this work are available at http://www.marcottelab.org/users/yungki.

### Method implementation

M1 and M2 were implemented by downloading and modifying the source code from the authors' websites, respectively. M3 and M4 were implemented on my own using the libsvm package [66]. The integrity of the M4 implementation was verified by correspondence with the authors.

Given that the number of PPIs in the cell is expected to be much smaller than that of possible protein-protein pairings, I initially tried to use negative PPI data of size > 2*N*, where *N *is the size of the positive PPI data, for training purposes. This procedure did not always lead to enhanced performance compared to using negative data of size *N*. In addition, it required unacceptably long computational time for some methods. Thus, I used negative data of size *N *for all training purposes. However, for testing purposes, I used negative data of size 100*N *for physiologically meaningful benchmarking (see the Results and Discussion section).

### Consensus approaches

Two different consensus approaches were tried, an SVC with a linear kernel (the linear SVC) and an SVC with a Gaussian kernel (the Gaussian SVC). The mathematical details of SVC and the linear and Gaussian kernels can be found in machine learning textbooks such as [59]. Both were implemented using the libsvm package [66], with all the parameters set to default values. Unlike the four methods, consensus approaches required additional training using classification scores generated by the four methods as input vectors. Thus, cross-validation was applied in a two-stage manner to prevent double-training and ensure unbiased performance estimation. The detailed scheme is shown in Additional File 3. The two SVCs yielded similar results. Here, I only discuss the results obtained with the linear SVC.

### Performance measure

Performance of each prediction method was measured in 4-fold cross-validation unless otherwise noted. Due to prohibitively high computational expenses, 4-fold cross-validation was carried out instead of 10-fold cross-validation, a more popular choice. Two figures were computed for estimating prediction performance. One is AUC, a widely used figure in these settings. The other is recall-precision. Recall is TP/(TP + FN), and precision is TP/(TP + FP), where TP is the number of true positives (i.e. a protein pair known to interact predicted to interact), FN is that of false negatives (i.e. a protein pair known to interact predicted not to interact), and FP is that of false positives (i.e. a protein pair assumed not to interact predicted to interact). As a single figure summarizing full recall-precision analysis, I report precision values at 20% recall (P20R) in the Tables. This is for reasons of space. However, all statistical analyses were based on full recall-precision plots. *P *values for estimating the statistical significance of performance differences between pairs of prediction methods were computed using the Wilcoxon signed rank test and are available in the Additional File 1.

## Abbreviations

PPI: protein-protein interaction; SVC: support vector classifier; AUC: area under the receiver operating characteristic curve; P20R: precision value at 20% recall

## Authors' contributions

YP designed the project, carried it out and wrote the manuscript.

## Supplementary Material

Additional file 1 ***P*****values for estimating statistical significance of performance difference between pairs of different prediction methods.** Click here for file

Additional file 2 Complete results for the analysis of prediction results by the GO slims. Click here for file

Additional file 3 Detailed scheme for a two-stage 4-fold cross-validation for the consensus method. Click here for file
