# Supplementary material for: Critical assessment of sequence-based protein-protein interaction prediction methods that do not require homologous protein sequences
Source: BMC Bioinformatics. 2009 Dec 14;10:419. doi: 10.1186/1471-2105-10-419 (PMC2803199; doi:10.1186/1471-2105-10-419)
Supplement: Additional file 1 — Pvalues for estimating statistical significance of performance difference between pairs of different prediction methods. [file 1471-2105-10-419-S1.DOC]

***P* values for estimating statistical significance of performance difference between pairs of prediction methods.**

Table titles are of the format A_B_CD. It indicates that the performance of prediction methods M1 – M4 and C (the consensus approach described in the main manuscript), when trained with A and tested on B, is estimated in terms of C. The size of the negative data is D times that of the positive data.

*P* values in the tables are read as follows. “1.46e-11” in the second row and the third column of the first table means that the p value of the null hypothesis that M1 and M2 perform equally is 1.46 X 10^-11^ as computed by the Wilcoxon signed rank test. Thus, the alternative hypothesis is strongly favored that M1 significantly outperforms M2 (one-sided test).

YEAST_YEAST_ROC100

----------------------------------------------------------

M1 M2 M3 M4 C

----------------------------------------------------------

M1 --- 1.46e-11 9.09e-13 9.09e-13 1.00e+00

M2 1.00e+00 --- 1.46e-11 3.82e-04 1.00e+00

M3 1.00e+00 1.00e+00 --- 1.00e+00 1.00e+00

M4 1.00e+00 1.00e+00 9.09e-13 --- 1.00e+00

C 1.82e-12 1.46e-11 9.09e-13 9.09e-13 ---

----------------------------------------------------------

YEAST_YEAST_ROC10

----------------------------------------------------------

M1 M2 M3 M4 C

----------------------------------------------------------

M1 --- 1.46e-11 9.09e-13 9.09e-13 1.00e+00

M2 1.00e+00 --- 1.46e-11 1.56e-04 1.00e+00

M3 1.00e+00 1.00e+00 --- 1.00e+00 1.00e+00

M4 1.00e+00 1.00e+00 9.09e-13 --- 1.00e+00

C 1.82e-12 1.46e-11 9.09e-13 9.09e-13 ---

----------------------------------------------------------

YEAST_YEAST_RP100

----------------------------------------------------------

M1 M2 M3 M4 C

----------------------------------------------------------

M1 --- 9.80e-01 9.09e-13 9.09e-13 1.00e+00

M2 2.07e-02 --- 1.82e-12 1.82e-12 1.00e+00

M3 1.00e+00 1.00e+00 --- 1.00e+00 1.00e+00

M4 1.00e+00 1.00e+00 9.09e-13 --- 1.00e+00

C 9.09e-13 4.88e-05 9.09e-13 9.09e-13 ---

----------------------------------------------------------

YEAST_YEAST_RP10

----------------------------------------------------------

M1 M2 M3 M4 C

----------------------------------------------------------

M1 --- 9.51e-01 9.09e-13 9.09e-13 1.00e+00

M2 5.07e-02 --- 1.82e-12 1.82e-12 1.00e+00

M3 1.00e+00 1.00e+00 --- 1.00e+00 1.00e+00

M4 1.00e+00 1.00e+00 9.09e-13 --- 1.00e+00

C 9.09e-13 6.00e-11 9.09e-13 9.09e-13 ---

----------------------------------------------------------

YEAST_HUMAN_ROC100

----------------------------------------------------------

M1 M2 M3 M4 C

----------------------------------------------------------

M1 --- 1.00e+00 9.09e-13 9.09e-13

M2 5.82e-11 --- 5.82e-11 5.82e-11

M3 1.00e+00 1.00e+00 --- 1.00e+00

M4 1.00e+00 1.00e+00 9.09e-13 ---

----------------------------------------------------------

YEAST_HUMAN_ROC10

----------------------------------------------------------

M1 M2 M3 M4 C

----------------------------------------------------------

M1 --- 1.00e+00 9.09e-13 9.09e-13

M2 5.82e-11 --- 5.82e-11 5.82e-11

M3 1.00e+00 1.00e+00 --- 1.00e+00

M4 1.00e+00 1.00e+00 9.09e-13 ---

----------------------------------------------------------

YEAST_HUMAN_RP100

----------------------------------------------------------

M1 M2 M3 M4 C

----------------------------------------------------------

M1 --- 1.00e+00 9.09e-13 9.09e-13

M2 7.28e-12 --- 7.28e-12 7.28e-12

M3 1.00e+00 1.00e+00 --- 1.00e+00

M4 1.00e+00 1.00e+00 9.09e-13 ---

----------------------------------------------------------

YEAST_HUMAN_RP10

----------------------------------------------------------

M1 M2 M3 M4 C

----------------------------------------------------------

M1 --- 1.00e+00 9.09e-13 9.09e-13

M2 7.28e-12 --- 7.28e-12 7.28e-12

M3 1.00e+00 1.00e+00 --- 1.00e+00

M4 1.00e+00 1.00e+00 9.09e-13 ---

----------------------------------------------------------

HUMAN_YEAST_ROC100

----------------------------------------------------------

M1 M2 M3 M4 C

----------------------------------------------------------

M1 --- 1.00e+00 9.09e-13 9.09e-13

M2 9.09e-13 --- 9.09e-13 9.09e-13

M3 1.00e+00 1.00e+00 --- 1.00e+00

M4 1.00e+00 1.00e+00 9.09e-13 ---

----------------------------------------------------------

HUMAN_YEAST_ROC10

----------------------------------------------------------

M1 M2 M3 M4 C

----------------------------------------------------------

M1 --- 1.00e+00 9.09e-13 9.09e-13

M2 9.09e-13 --- 9.09e-13 9.09e-13

M3 1.00e+00 1.00e+00 --- 1.00e+00

M4 1.00e+00 1.00e+00 9.09e-13 ---

----------------------------------------------------------

HUMAN_YEAST_RP100

----------------------------------------------------------

M1 M2 M3 M4 C

----------------------------------------------------------

M1 --- 1.00e+00 9.09e-13 9.09e-13

M2 9.09e-13 --- 9.09e-13 9.09e-13

M3 1.00e+00 1.00e+00 --- 1.00e+00

M4 1.00e+00 1.00e+00 1.82e-12 ---

----------------------------------------------------------

HUMAN_YEAST_RP10

----------------------------------------------------------

M1 M2 M3 M4 C

----------------------------------------------------------

M1 --- 1.00e+00 9.09e-13 9.09e-13

M2 9.09e-13 --- 9.09e-13 9.09e-13

M3 1.00e+00 1.00e+00 --- 1.00e+00

M4 1.00e+00 1.00e+00 9.09e-13 ---

----------------------------------------------------------

HUMAN_HUMAN_ROC100

----------------------------------------------------------

M1 M2 M3 M4 C

----------------------------------------------------------

M1 --- 9.09e-13 9.09e-13 9.09e-13 1.00e+00

M2 1.00e+00 --- 9.09e-13 1.00e+00 1.00e+00

M3 1.00e+00 1.00e+00 --- 1.00e+00 1.00e+00

M4 1.00e+00 9.09e-13 9.09e-13 --- 1.00e+00

C 9.09e-13 9.09e-13 9.09e-13 9.09e-13 ---

----------------------------------------------------------

HUMAN_HUMAN_ROC10

----------------------------------------------------------

M1 M2 M3 M4 C

----------------------------------------------------------

M1 --- 9.09e-13 9.09e-13 9.09e-13 1.00e+00

M2 1.00e+00 --- 9.09e-13 1.00e+00 1.00e+00

M3 1.00e+00 1.00e+00 --- 1.00e+00 1.00e+00

M4 1.00e+00 9.09e-13 9.09e-13 --- 1.00e+00

C 9.09e-13 9.09e-13 9.09e-13 9.09e-13 ---

----------------------------------------------------------

HUMAN_HUMAN_RP100

----------------------------------------------------------

M1 M2 M3 M4 C

----------------------------------------------------------

M1 --- 9.63e-01 9.09e-13 9.09e-13 1.00e+00

M2 3.85e-02 --- 9.09e-13 2.69e-04 1.00e+00

M3 1.00e+00 1.00e+00 --- 1.00e+00 1.00e+00

M4 1.00e+00 1.00e+00 9.09e-13 --- 1.00e+00

C 9.09e-13 9.09e-13 9.09e-13 9.09e-13 ---

----------------------------------------------------------

HUMAN_HUMAN_RP10

----------------------------------------------------------

M1 M2 M3 M4 C

----------------------------------------------------------

M1 --- 9.06e-01 9.09e-13 9.09e-13 1.00e+00

M2 9.61e-02 --- 9.09e-13 5.24e-04 1.00e+00

M3 1.00e+00 1.00e+00 --- 1.00e+00 1.00e+00

M4 1.00e+00 1.00e+00 9.09e-13 --- 1.00e+00

C 9.09e-13 9.09e-13 9.09e-13 9.09e-13 ---

----------------------------------------------------------

COMBINED_YEAST_ROC100

----------------------------------------------------------

M1 M2 M3 M4 C

----------------------------------------------------------

M1 --- 8.36e-06 9.09e-13 9.09e-13 1.00e+00

M2 1.00e+00 --- 9.09e-13 9.09e-13 1.00e+00

M3 1.00e+00 1.00e+00 --- 1.00e+00 1.00e+00

M4 1.00e+00 1.00e+00 9.09e-13 --- 1.00e+00

C 9.09e-13 9.09e-13 9.09e-13 9.09e-13 ---

----------------------------------------------------------

COMBINED_YEAST_ROC10

----------------------------------------------------------

M1 M2 M3 M4 C

----------------------------------------------------------

M1 --- 5.81e-07 9.09e-13 9.09e-13 1.00e+00

M2 1.00e+00 --- 9.09e-13 9.09e-13 1.00e+00

M3 1.00e+00 1.00e+00 --- 1.00e+00 1.00e+00

M4 1.00e+00 1.00e+00 9.09e-13 --- 1.00e+00

C 8.00e-11 9.09e-13 9.09e-13 9.09e-13 ---

----------------------------------------------------------

COMBINED_YEAST_RP100

----------------------------------------------------------

M1 M2 M3 M4 C

----------------------------------------------------------

M1 --- 1.00e+00 9.09e-13 9.09e-13 1.00e+00

M2 9.75e-06 --- 9.09e-13 9.09e-13 1.00e+00

M3 1.00e+00 1.00e+00 --- 1.00e+00 1.00e+00

M4 1.00e+00 1.00e+00 9.09e-13 --- 1.00e+00

C 9.09e-13 9.09e-13 9.09e-13 9.09e-13 ---

----------------------------------------------------------

COMBINED_YEAST_RP10

----------------------------------------------------------

M1 M2 M3 M4 C

----------------------------------------------------------

M1 --- 1.00e+00 9.09e-13 9.09e-13 1.00e+00

M2 4.72e-05 --- 9.09e-13 9.09e-13 1.00e+00

M3 1.00e+00 1.00e+00 --- 1.00e+00 1.00e+00

M4 1.00e+00 1.00e+00 9.09e-13 --- 1.00e+00

C 9.09e-13 9.09e-13 9.09e-13 9.09e-13 ---

----------------------------------------------------------

COMBINED_HUMAN_ROC100

----------------------------------------------------------

M1 M2 M3 M4 C

----------------------------------------------------------

M1 --- 9.09e-13 9.09e-13 9.09e-13 1.00e+00

M2 1.00e+00 --- 9.09e-13 1.00e+00 1.00e+00

M3 1.00e+00 1.00e+00 --- 1.00e+00 1.00e+00

M4 1.00e+00 1.27e-11 9.09e-13 --- 1.00e+00

C 9.09e-13 9.09e-13 9.09e-13 9.09e-13 ---

----------------------------------------------------------

COMBINED_HUMAN_ROC10

----------------------------------------------------------

M1 M2 M3 M4 C

----------------------------------------------------------

M1 --- 9.09e-13 9.09e-13 9.09e-13 1.00e+00

M2 1.00e+00 --- 9.09e-13 1.00e+00 1.00e+00

M3 1.00e+00 1.00e+00 --- 1.00e+00 1.00e+00

M4 1.00e+00 1.27e-11 9.09e-13 --- 1.00e+00

C 9.09e-13 9.09e-13 9.09e-13 9.09e-13 ---

----------------------------------------------------------

COMBINED_HUMAN_RP100

----------------------------------------------------------

M1 M2 M3 M4 C

----------------------------------------------------------

M1 --- 9.88e-01 9.09e-13 9.09e-13 1.00e+00

M2 1.25e-02 --- 9.09e-13 1.14e-05 1.00e+00

M3 1.00e+00 1.00e+00 --- 1.00e+00 1.00e+00

M4 1.00e+00 1.00e+00 9.09e-13 --- 1.00e+00

C 9.09e-13 9.09e-13 9.09e-13 9.09e-13 ---

----------------------------------------------------------

COMBINED_HUMAN_RP10

----------------------------------------------------------

M1 M2 M3 M4 C

----------------------------------------------------------

M1 --- 9.71e-01 9.09e-13 9.09e-13 1.00e+00

M2 3.01e-02 --- 9.09e-13 2.37e-05 1.00e+00

M3 1.00e+00 1.00e+00 --- 1.00e+00 1.00e+00

M4 1.00e+00 1.00e+00 9.09e-13 --- 1.00e+00

C 9.09e-13 9.09e-13 9.09e-13 9.09e-13 ---

----------------------------------------------------------

COMBINED_COMBINED_ROC100

----------------------------------------------------------

M1 M2 M3 M4 C

----------------------------------------------------------

M1 --- 9.09e-13 9.09e-13 9.09e-13 1.00e+00

M2 1.00e+00 --- 9.09e-13 1.00e+00 1.00e+00

M3 1.00e+00 1.00e+00 --- 1.00e+00 1.00e+00

M4 1.00e+00 1.57e-07 9.09e-13 --- 1.00e+00

C 9.09e-13 9.09e-13 9.09e-13 9.09e-13 ---

----------------------------------------------------------

COMBINED_COMBINED_ROC10

----------------------------------------------------------

M1 M2 M3 M4 C

----------------------------------------------------------

M1 --- 9.09e-13 9.09e-13 9.09e-13 1.00e+00

M2 1.00e+00 --- 9.09e-13 1.00e+00 1.00e+00

M3 1.00e+00 1.00e+00 --- 1.00e+00 1.00e+00

M4 1.00e+00 1.57e-07 9.09e-13 --- 1.00e+00

C 9.09e-13 9.09e-13 9.09e-13 9.09e-13 ---

----------------------------------------------------------

COMBINED_COMBINED_RP100

----------------------------------------------------------

M1 M2 M3 M4 C

----------------------------------------------------------

M1 --- 9.88e-01 9.09e-13 9.09e-13 1.00e+00

M2 1.20e-02 --- 9.09e-13 1.34e-06 1.00e+00

M3 1.00e+00 1.00e+00 --- 1.00e+00 1.00e+00

M4 1.00e+00 1.00e+00 9.09e-13 --- 1.00e+00

C 9.09e-13 9.09e-13 9.09e-13 9.09e-13 ---

----------------------------------------------------------

COMBINED_COMBINED_RP10

----------------------------------------------------------

M1 M2 M3 M4 C

----------------------------------------------------------

M1 --- 9.72e-01 9.09e-13 9.09e-13 1.00e+00

M2 2.92e-02 --- 9.09e-13 2.26e-06 1.00e+00

M3 1.00e+00 1.00e+00 --- 1.00e+00 1.00e+00

M4 1.00e+00 1.00e+00 9.09e-13 --- 1.00e+00

C 9.09e-13 9.09e-13 9.09e-13 9.09e-13 ---

----------------------------------------------------------
