# Supplementary material for: Critical assessment of sequence-based protein-protein interaction prediction methods that do not require homologous protein sequences
Source: BMC Bioinformatics. 2009 Dec 14;10:419. doi: 10.1186/1471-2105-10-419 (PMC2803199; doi:10.1186/1471-2105-10-419)
Supplement: Additional file 2 — Complete results for the analysis of prediction results by the GO slims. [file 1471-2105-10-419-S2.PDF]

YEAST results sorted according to AUC

	GO term	# of cases	Best method	AUC	Best method	P20R	Explanation of GO term
1	GO:0005198	39513	Consensus	0.90	Consensus	0.92	structural molecule activity
2	GO:0007124	9192	Consensus	0.89	M2	0.44	pseudohyphal growth
3	GO:0006997	10093	Consensus	0.89	Consensus	0.97	nucleus organization
4	GO:0007047	18668	Consensus	0.89	M2	0.83	cell wall organization
5	GO:0005215	44019	Consensus	0.89	M2	0.62	transporter activity
6	GO:0019725	16801	Consensus	0.88	Consensus	0.33	cellular homeostasis
7	GO:0042254	44304	Consensus	0.87	Consensus	0.95	ribosome biogenesis
8	GO:0005773	25446	Consensus	0.87	M2	0.32	vacuole
9	GO:0005618	8689	M1	0.87	M2	1.00	cell wall
10	GO:0000910	17082	Consensus	0.87	M2	0.72	cytokinesis
11	GO:0030435	16477	Consensus	0.87	M2	0.51	sporulation resulting in formation of a cellular spore
12	GO:0016874	22332	Consensus	0.87	Consensus	0.32	ligase activity
13	GO:0004386	10148	Consensus	0.87	Consensus	0.15	helicase activity
14	GO:0007114	12912	Consensus	0.87	M2	0.79	cell budding
15	GO:0012505	46604	Consensus	0.86	M2	0.91	endomembrane system
16	GO:0005794	27472	Consensus	0.86	M2	0.76	Golgi apparatus
17	GO:0006412	42152	Consensus	0.86	M2	0.29	translation
18	GO:0016787	92927	Consensus	0.86	M2	0.62	hydrolase activity
19	GO:0000746	16486	Consensus	0.86	M2	0.42	conjugation
20	GO:0006810	121074	Consensus	0.86	M2	0.46	transport
21	GO:0003723	30748	Consensus	0.86	M2	0.75	RNA binding
22	GO:0032989	22264	Consensus	0.85	M2	0.75	cellular component morphogenesis
23	GO:0007033	5872	Consensus	0.85	M2	0.38	vacuole organization
24	GO:0042221	50737	Consensus	0.85	Consensus	0.27	response to chemical stimulus
25	GO:0005624	21509	Consensus	0.85	Consensus	0.26	membrane fraction
26	GO:0016023	15191	Consensus	0.85	M2	0.75	cytoplasmic membrane-bounded vesicle
27	GO:0005938	17461	Consensus	0.85	M2	0.71	cell cortex
28	GO:0005886	29530	Consensus	0.85	Consensus	0.11	plasma membrane
29	GO:0016070	142192	Consensus	0.85	M2	0.31	RNA metabolic process
30	GO:0005634	218939	Consensus	0.85	Consensus	0.34	nucleus
31	GO:0005933	24507	Consensus	0.85	M2	0.65	cellular bud
32	GO:0044255	29992	Consensus	0.85	M2	0.14	cellular lipid metabolic process
33	GO:0030427	31539	Consensus	0.85	M2	0.51	site of polarized growth
34	GO:0003677	45694	Consensus	0.85	Consensus	0.31	DNA binding
35	GO:0016020	114517	Consensus	0.84	M2	0.68	membrane
36	GO:0006464	70314	Consensus	0.84	Consensus	0.27	protein modification process
37	GO:0044257	28527	Consensus	0.84	M2	0.34	cellular protein catabolic process
38	GO:0016044	35037	Consensus	0.84	M2	0.42	membrane organization
39	GO:0005739	120843	Consensus	0.84	M2	0.30	mitochondrion

40	GO:0005515	76575	Consensus	0.84	M2	0.36	protein binding
41	GO:0003774	2399	Consensus	0.84	M2	0.75	motor activity
42	GO:0051276	54607	Consensus	0.84	Consensus	0.23	chromosome organization
43	GO:0008289	10690	Consensus	0.84	M2	0.92	lipid binding
44	GO:0004672	17648	Consensus	0.84	M2	0.40	protein kinase activity
45	GO:0005737	283867	Consensus	0.84	Consensus	0.33	cytoplasm
46	GO:0032196	6625	Consensus	0.84	Consensus	0.51	transposition
47	GO:0016192	48693	Consensus	0.84	M2	0.33	vesicle-mediated transport
48	GO:0005694	48196	Consensus	0.84	Consensus	0.30	chromosome
49	GO:0008233	14819	Consensus	0.84	M2	0.80	peptidase activity
50	GO:0030528	47284	Consensus	0.83	Consensus	0.17	transcription regulator activity
51	GO:0007059	21592	Consensus	0.83	M2	0.54	chromosome segregation
52	GO:0004871	5614	Consensus	0.83	M2	0.37	signal transducer activity
53	GO:0006350	84696	Consensus	0.83	Consensus	0.21	transcription
54	GO:0007010	32379	Consensus	0.83	Consensus	0.30	cytoskeleton organization
55	GO:0005840	36169	Consensus	0.83	M2	0.36	ribosome
56	GO:0006259	54363	Consensus	0.83	M2	0.25	DNA metabolic process
57	GO:0005730	36321	Consensus	0.83	M2	0.62	nucleolus
58	GO:0007049	72933	Consensus	0.83	M2	0.31	cell cycle
59	GO:0030234	30937	Consensus	0.83	Consensus	0.52	enzyme regulator activity
60	GO:0070271	19752	Consensus	0.83	M2	0.30	protein complex biogenesis
61	GO:0006950	71093	Consensus	0.83	Consensus	0.28	response to stress
62	GO:0003674	218249	Consensus	0.83	Consensus	0.07	molecular_function
63	GO:0045182	6716	Consensus	0.82	Consensus	0.14	translation regulator activity
64	GO:0016050	9343	Consensus	0.82	M2	0.49	vesicle organization
65	GO:0005856	31614	Consensus	0.82	Consensus	0.39	cytoskeleton
66	GO:0005783	46376	Consensus	0.82	M2	0.21	endoplasmic reticulum
67	GO:0016740	79604	Consensus	0.82	Consensus	0.22	transferase activity
68	GO:0007165	32994	Consensus	0.82	M2	0.27	signal transduction
69	GO:0007005	36372	Consensus	0.80	M2	0.35	mitochondrion organization
70	GO:0006091	21768	Consensus	0.80	M2	0.19	generation of precursor metabolites and energy
71	GO:0005815	11381	Consensus	0.80	Consensus	0.17	microtubule organizing center
72	GO:0016779	10915	Consensus	0.80	Consensus	0.18	nucleotidyltransferase activity
73	GO:0045333	11860	Consensus	0.80	M1	0.06	cellular respiration
74	GO:0005777	8440	Consensus	0.79	Consensus	0.06	peroxisome
75	GO:0008150	145550	Consensus	0.79	Consensus	0.03	biological_process
76	GO:0044262	27231	Consensus	0.79	M2	0.23	cellular carbohydrate metabolic process
77	GO:0005576	1728	M1	0.79	M3	0.07	extracellular region
78	GO:0006457	9885	Consensus	0.78	Consensus	0.12	protein folding
79	GO:0007126	21328	Consensus	0.78	M2	0.14	meiosis
80	GO:0005575	97107	Consensus	0.77	Consensus	0.02	cellular_component

81	GO:0006519	30652	M1	0.77	Consensus	0.11	cellular amino acid and derivative metabolic process
82	GO:0007031	7290	Consensus	0.76	Consensus	0.07	peroxisome organization
83	GO:0016853	7165	Consensus	0.76	M1	0.03	isomerase activity
84	GO:0046483	19491	Consensus	0.76	M2	0.45	heterocycle metabolic process
85	GO:0005740	35294	Consensus	0.74	M2	0.23	mitochondrial envelope
86	GO:0004721	6340	Consensus	0.74	M2	0.50	phosphoprotein phosphatase activity
87	GO:0016829	10313	Consensus	0.72	Consensus	0.03	lyase activity
88	GO:0006725	9209	Consensus	0.72	M1	0.08	cellular aromatic compound metabolic process
89	GO:0051186	17880	Consensus	0.70	Consensus	0.04	cofactor metabolic process
90	GO:0006766	9004	M1	0.67	M2	0.01	vitamin metabolic process
91	GO:0016491	28139	Consensus	0.63	M3	0.02	oxidoreductase activity

YEAST results sorted according to P20R

	GO term	# of cases	Best method	AUC	Best method	P20R	Explanation of GO term
1	GO:0005618	8689	M1	0.87	M2	1.00	cell wall
2	GO:0006997	10093	Consensus	0.89	Consensus	0.97	nucleus organization
3	GO:0042254	44304	Consensus	0.87	Consensus	0.95	ribosome biogenesis
4	GO:0005198	39513	Consensus	0.90	Consensus	0.92	structural molecule activity
5	GO:0008289	10690	Consensus	0.84	M2	0.92	lipid binding
6	GO:0012505	46604	Consensus	0.86	M2	0.91	endomembrane system
7	GO:0007047	18668	Consensus	0.89	M2	0.83	cell wall organization
8	GO:0008233	14819	Consensus	0.84	M2	0.80	peptidase activity
9	GO:0007114	12912	Consensus	0.87	M2	0.79	cell budding
10	GO:0005794	27472	Consensus	0.86	M2	0.76	Golgi apparatus
11	GO:0032989	22264	Consensus	0.85	M2	0.75	cellular component morphogenesis
12	GO:0003774	2399	Consensus	0.84	M2	0.75	motor activity
13	GO:0003723	30748	Consensus	0.86	M2	0.75	RNA binding
14	GO:0016023	15191	Consensus	0.85	M2	0.75	cytoplasmic membrane-bounded vesicle
15	GO:0000910	17082	Consensus	0.87	M2	0.72	cytokinesis
16	GO:0005938	17461	Consensus	0.85	M2	0.71	cell cortex
17	GO:0016020	114517	Consensus	0.84	M2	0.68	membrane
18	GO:0005933	24507	Consensus	0.85	M2	0.65	cellular bud
19	GO:0005730	36321	Consensus	0.83	M2	0.62	nucleolus
20	GO:0016787	92927	Consensus	0.86	M2	0.62	hydrolase activity
21	GO:0005215	44019	Consensus	0.89	M2	0.62	transporter activity
22	GO:0007059	21592	Consensus	0.83	M2	0.54	chromosome segregation
23	GO:0030234	30937	Consensus	0.83	Consensus	0.52	enzyme regulator activity
24	GO:0030435	16477	Consensus	0.87	M2	0.51	sporulation resulting in formation of a cellular spore

25	GO:0032196	6625	Consensus	0.84	Consensus	0.51	transposition
26	GO:0030427	31539	Consensus	0.85	M2	0.51	site of polarized growth
27	GO:0004721	6340	Consensus	0.74	M2	0.50	phosphoprotein phosphatase activity
28	GO:0016050	9343	Consensus	0.82	M2	0.49	vesicle organization
29	GO:0006810	121074	Consensus	0.86	M2	0.46	transport
30	GO:0046483	19491	Consensus	0.76	M2	0.45	heterocycle metabolic process
31	GO:0007124	9192	Consensus	0.89	M2	0.44	pseudohyphal growth
32	GO:0000746	16486	Consensus	0.86	M2	0.42	conjugation
33	GO:0016044	35037	Consensus	0.84	M2	0.42	membrane organization
34	GO:0004672	17648	Consensus	0.84	M2	0.40	protein kinase activity
35	GO:0005856	31614	Consensus	0.82	Consensus	0.39	cytoskeleton
36	GO:0007033	5872	Consensus	0.85	M2	0.38	vacuole organization
37	GO:0004871	5614	Consensus	0.83	M2	0.37	signal transducer activity
38	GO:0005840	36169	Consensus	0.83	M2	0.36	ribosome
39	GO:0005515	76575	Consensus	0.84	M2	0.36	protein binding
40	GO:0007005	36372	Consensus	0.80	M2	0.35	mitochondrion organization
41	GO:0005634	218939	Consensus	0.85	Consensus	0.34	nucleus
42	GO:0044257	28527	Consensus	0.84	M2	0.34	cellular protein catabolic process
43	GO:0005737	283867	Consensus	0.84	Consensus	0.33	cytoplasm
44	GO:0019725	16801	Consensus	0.88	Consensus	0.33	cellular homeostasis
45	GO:0016192	48693	Consensus	0.84	M2	0.33	vesicle-mediated transport
46	GO:0005773	25446	Consensus	0.87	M2	0.32	vacuole
47	GO:0016874	22332	Consensus	0.87	Consensus	0.32	ligase activity
48	GO:0003677	45694	Consensus	0.85	Consensus	0.31	DNA binding
49	GO:0016070	142192	Consensus	0.85	M2	0.31	RNA metabolic process
50	GO:0007049	72933	Consensus	0.83	M2	0.31	cell cycle
51	GO:0070271	19752	Consensus	0.83	M2	0.30	protein complex biogenesis
52	GO:0005739	120843	Consensus	0.84	M2	0.30	mitochondrion
53	GO:0007010	32379	Consensus	0.83	Consensus	0.30	cytoskeleton organization
54	GO:0005694	48196	Consensus	0.84	Consensus	0.30	chromosome
55	GO:0006412	42152	Consensus	0.86	M2	0.29	translation
56	GO:0006950	71093	Consensus	0.83	Consensus	0.28	response to stress
57	GO:0042221	50737	Consensus	0.85	Consensus	0.27	response to chemical stimulus
58	GO:0007165	32994	Consensus	0.82	M2	0.27	signal transduction
59	GO:0006464	70314	Consensus	0.84	Consensus	0.27	protein modification process
60	GO:0005624	21509	Consensus	0.85	Consensus	0.26	membrane fraction
61	GO:0006259	54363	Consensus	0.83	M2	0.25	DNA metabolic process
62	GO:0044262	27231	Consensus	0.79	M2	0.23	cellular carbohydrate metabolic process
63	GO:0051276	54607	Consensus	0.84	Consensus	0.23	chromosome organization
64	GO:0005740	35294	Consensus	0.74	M2	0.23	mitochondrial envelope
65	GO:0016740	79604	Consensus	0.82	Consensus	0.22	transferase activity
66	GO:0005783	46376	Consensus	0.82	M2	0.21	endoplasmic reticulum

67	GO:0006350	84696	Consensus	0.83	Consensus	0.21	transcription
68	GO:0006091	21768	Consensus	0.80	M2	0.19	generation of precursor metabolites and energy
69	GO:0016779	10915	Consensus	0.80	Consensus	0.18	nucleotidyltransferase activity
70	GO:0030528	47284	Consensus	0.83	Consensus	0.17	transcription regulator activity
71	GO:0005815	11381	Consensus	0.80	Consensus	0.17	microtubule organizing center
72	GO:0004386	10148	Consensus	0.87	Consensus	0.15	helicase activity
73	GO:0044255	29992	Consensus	0.85	M2	0.14	cellular lipid metabolic process
74	GO:0007126	21328	Consensus	0.78	M2	0.14	meiosis
75	GO:0045182	6716	Consensus	0.82	Consensus	0.14	translation regulator activity
76	GO:0006457	9885	Consensus	0.78	Consensus	0.12	protein folding
77	GO:0006519	30652	M1	0.77	Consensus	0.11	cellular amino acid and derivative metabolic process
78	GO:0005886	29530	Consensus	0.85	Consensus	0.11	plasma membrane
79	GO:0006725	9209	Consensus	0.72	M1	0.08	cellular aromatic compound metabolic process
80	GO:0007031	7290	Consensus	0.76	Consensus	0.07	peroxisome organization
81	GO:0005576	1728	M1	0.79	M3	0.07	extracellular region
82	GO:0003674	218249	Consensus	0.83	Consensus	0.07	molecular_function
83	GO:0045333	11860	Consensus	0.80	M1	0.06	cellular respiration
84	GO:0005777	8440	Consensus	0.79	Consensus	0.06	peroxisome
85	GO:0051186	17880	Consensus	0.70	Consensus	0.04	cofactor metabolic process
86	GO:0016853	7165	Consensus	0.76	M1	0.03	isomerase activity
87	GO:0016829	10313	Consensus	0.72	Consensus	0.03	lyase activity
88	GO:0008150	145550	Consensus	0.79	Consensus	0.03	biological_process
89	GO:0016491	28139	Consensus	0.63	M3	0.02	oxidoreductase activity
90	GO:0005575	97107	Consensus	0.77	Consensus	0.02	cellular_component
91	GO:0006766	9004	M1	0.67	M2	0.01	vitamin metabolic process

HUMAN results sorted according to AUC

	GO term	# of cases	Best method	AUC	Best method	P20R	Explanation of GO term
1	GO:0008907	245	Consensus	1.00	M2	1.00	integrase activity
2	GO:0004871	71939	Consensus	0.92	Consensus	0.91	signal transducer activity
3	GO:0051704	88280	Consensus	0.92	Consensus	0.84	multi-organism process
4	GO:0008219	98990	Consensus	0.92	Consensus	0.86	cell death
5	GO:0016740	244001	Consensus	0.91	Consensus	0.84	transferase activity
6	GO:0030528	236038	Consensus	0.91	Consensus	0.78	transcription regulator activity
7	GO:0005694	72648	Consensus	0.91	Consensus	0.66	chromosome
8	GO:0016301	110554	Consensus	0.91	Consensus	0.88	kinase activity

9	GO:0004872	208752	Consensus	0.91	Consensus	0.88	receptor activity
10	GO:0030154	178593	Consensus	0.91	Consensus	0.87	cell differentiation
11	GO:0007154	458362	Consensus	0.91	Consensus	0.82	cell communication
12	GO:0016874	65777	Consensus	0.91	Consensus	0.57	ligase activity
13	GO:0007275	320661	Consensus	0.91	Consensus	0.80	multicellular organismal development
14	GO:0003676	407096	Consensus	0.91	Consensus	0.67	nucleic acid binding
15	GO:0050896	365227	Consensus	0.91	Consensus	0.81	response to stimulus
16	GO:0008150	372497	Consensus	0.91	Consensus	0.79	biological_process
17	GO:0005488	898947	Consensus	0.90	Consensus	0.73	binding
18	GO:0016020	885962	Consensus	0.90	Consensus	0.74	membrane
19	GO:0008152	559693	Consensus	0.90	Consensus	0.77	metabolic process
20	GO:0005622	394638	Consensus	0.90	Consensus	0.69	intracellular
21	GO:0007610	33647	Consensus	0.90	Consensus	0.82	behavior
22	GO:0043170	487875	Consensus	0.90	Consensus	0.75	macromolecule metabolic process
23	GO:0009056	210214	Consensus	0.90	Consensus	0.72	catabolic process
24	GO:0005634	714744	Consensus	0.90	Consensus	0.69	nucleus
25	GO:0050789	697322	Consensus	0.90	Consensus	0.76	regulation of biological process
26	GO:0005737	965262	Consensus	0.90	Consensus	0.71	cytoplasm
27	GO:0032501	154712	Consensus	0.90	Consensus	0.82	multicellular organismal process
28	GO:0009405	1017	Consensus	0.90	M2	1.00	pathogenesis
29	GO:0006810	348593	Consensus	0.90	Consensus	0.68	transport
30	GO:0009987	649957	Consensus	0.90	Consensus	0.74	cellular process
31	GO:0005575	630872	Consensus	0.89	Consensus	0.71	cellular_component
32	GO:0015267	7183	Consensus	0.89	M2	0.85	channel activity
33	GO:0016787	313025	Consensus	0.89	Consensus	0.64	hydrolase activity
34	GO:0006928	52075	Consensus	0.89	Consensus	0.87	cell motion
35	GO:0005623	202585	Consensus	0.89	Consensus	0.76	cell
36	GO:0005515	1058738	Consensus	0.89	Consensus	0.70	protein binding
37	GO:0030234	131962	Consensus	0.89	Consensus	0.70	enzyme regulator activity
38	GO:0004386	25817	Consensus	0.89	Consensus	0.58	helicase activity
39	GO:0005578	50953	Consensus	0.89	Consensus	0.79	proteinaceous extracellular matrix
40	GO:0005215	86473	Consensus	0.89	Consensus	0.57	transporter activity
41	GO:0009986	44472	Consensus	0.89	Consensus	0.83	cell surface
42	GO:0043062	15500	Consensus	0.89	Consensus	0.80	extracellular structure organization
43	GO:0006139	252980	Consensus	0.89	Consensus	0.57	nucleobase, nucleoside, nucleotide and nucleic acid metabolic process
44	GO:0005198	87092	Consensus	0.88	Consensus	0.68	structural molecule activity
45	GO:0003674	111593	Consensus	0.88	Consensus	0.39	molecular_function
46	GO:0045182	18978	Consensus	0.88	Consensus	0.27	translation regulator activity
47	GO:0016853	22610	Consensus	0.88	Consensus	0.40	isomerase activity
48	GO:0005576	283486	Consensus	0.87	Consensus	0.67	extracellular region
49	GO:0005615	108395	Consensus	0.87	M2	0.82	extracellular space

50	GO:0015075	85927	Consensus	0.87	Consensus	0.59	ion transmembrane transporter activity
51	GO:0003824	29099	Consensus	0.87	Consensus	0.31	catalytic activity
52	GO:0046903	34882	Consensus	0.86	M2	0.63	secretion
53	GO:0009058	147661	Consensus	0.86	Consensus	0.65	biosynthetic process
54	GO:0016491	103139	Consensus	0.86	Consensus	0.25	oxidoreductase activity
55	GO:0008565	13696	Consensus	0.85	Consensus	0.43	protein transporter activity
56	GO:0006519	44750	Consensus	0.85	Consensus	0.82	cellular amino acid and derivative metabolic process
57	GO:0003774	19797	Consensus	0.83	Consensus	0.23	motor activity
58	GO:0006944	8531	Consensus	0.83	Consensus	0.62	membrane fusion
59	GO:0009055	33126	Consensus	0.81	Consensus	0.11	electron carrier activity
60	GO:0016829	24145	Consensus	0.78	Consensus	0.23	lyase activity
61	GO:0016209	6685	Consensus	0.77	Consensus	0.21	antioxidant activity
62	GO:0030312	482	M1	0.69	M2	0.00	external encapsulating structure

HUMAN results sorted according to P20R

	GO term	# of cases	Best method	AUC	Best method	P20R	Explanation of GO term
1	GO:0009405	1017	Consensus	0.90	M2	1.00	pathogenesis
2	GO:0008907	245	Consensus	1.00	M2	1.00	integrase activity
3	GO:0004871	71939	Consensus	0.92	Consensus	0.91	signal transducer activity
4	GO:0004872	208752	Consensus	0.91	Consensus	0.88	receptor activity
5	GO:0016301	110554	Consensus	0.91	Consensus	0.88	kinase activity
6	GO:0006928	52075	Consensus	0.89	Consensus	0.87	cell motion
7	GO:0030154	178593	Consensus	0.91	Consensus	0.87	cell differentiation
8	GO:0008219	98990	Consensus	0.92	Consensus	0.86	cell death
9	GO:0015267	7183	Consensus	0.89	M2	0.85	channel activity
10	GO:0051704	88280	Consensus	0.92	Consensus	0.84	multi-organism process
11	GO:0016740	244001	Consensus	0.91	Consensus	0.84	transferase activity
12	GO:0009986	44472	Consensus	0.89	Consensus	0.83	cell surface
13	GO:0032501	154712	Consensus	0.90	Consensus	0.82	multicellular organismal process
14	GO:0006519	44750	Consensus	0.85	Consensus	0.82	cellular amino acid and derivative metabolic process
15	GO:0007610	33647	Consensus	0.90	Consensus	0.82	behavior
16	GO:0005615	108395	Consensus	0.87	M2	0.82	extracellular space
17	GO:0007154	458362	Consensus	0.91	Consensus	0.82	cell communication
18	GO:0050896	365227	Consensus	0.91	Consensus	0.81	response to stimulus
19	GO:0007275	320661	Consensus	0.91	Consensus	0.80	multicellular organismal development
20	GO:0043062	15500	Consensus	0.89	Consensus	0.80	extracellular structure organization
21	GO:0008150	372497	Consensus	0.91	Consensus	0.79	biological_process
22	GO:0005578	50953	Consensus	0.89	Consensus	0.79	proteinaceous extracellular matrix
23	GO:0030528	236038	Consensus	0.91	Consensus	0.78	transcription regulator activity

24	GO:0008152	559693	Consensus	0.90	Consensus	0.77	metabolic process
25	GO:0005623	202585	Consensus	0.89	Consensus	0.76	cell
26	GO:0050789	697322	Consensus	0.90	Consensus	0.76	regulation of biological process
27	GO:0043170	487875	Consensus	0.90	Consensus	0.75	macromolecule metabolic process
28	GO:0016020	885962	Consensus	0.90	Consensus	0.74	membrane
29	GO:0009987	649957	Consensus	0.90	Consensus	0.74	cellular process
30	GO:0005488	898947	Consensus	0.90	Consensus	0.73	binding
31	GO:0009056	210214	Consensus	0.90	Consensus	0.72	catabolic process
32	GO:0005575	630872	Consensus	0.89	Consensus	0.71	cellular_component
33	GO:0005737	965262	Consensus	0.90	Consensus	0.71	cytoplasm
34	GO:0005515	1058738	Consensus	0.89	Consensus	0.70	protein binding
35	GO:0030234	131962	Consensus	0.89	Consensus	0.70	enzyme regulator activity
36	GO:0005622	394638	Consensus	0.90	Consensus	0.69	intracellular
37	GO:0005634	714744	Consensus	0.90	Consensus	0.69	nucleus
38	GO:0005198	87092	Consensus	0.88	Consensus	0.68	structural molecule activity
39	GO:0006810	348593	Consensus	0.90	Consensus	0.68	transport
40	GO:0005576	283486	Consensus	0.87	Consensus	0.67	extracellular region
41	GO:0003676	407096	Consensus	0.91	Consensus	0.67	nucleic acid binding
42	GO:0005694	72648	Consensus	0.91	Consensus	0.66	chromosome
43	GO:0009058	147661	Consensus	0.86	Consensus	0.65	biosynthetic process
44	GO:0016787	313025	Consensus	0.89	Consensus	0.64	hydrolase activity
45	GO:0046903	34882	Consensus	0.86	M2	0.63	secretion
46	GO:0006944	8531	Consensus	0.83	Consensus	0.62	membrane fusion
47	GO:0015075	85927	Consensus	0.87	Consensus	0.59	ion transmembrane transporter activity
48	GO:0004386	25817	Consensus	0.89	Consensus	0.58	helicase activity
49	GO:0006139	252980	Consensus	0.89	Consensus	0.57	nucleobase, nucleoside, nucleotide and nucleic acid metabolic process
50	GO:0016874	65777	Consensus	0.91	Consensus	0.57	ligase activity
51	GO:0005215	86473	Consensus	0.89	Consensus	0.57	transporter activity
52	GO:0008565	13696	Consensus	0.85	Consensus	0.43	protein transporter activity
53	GO:0016853	22610	Consensus	0.88	Consensus	0.40	isomerase activity
54	GO:0003674	111593	Consensus	0.88	Consensus	0.39	molecular_function
55	GO:0003824	29099	Consensus	0.87	Consensus	0.31	catalytic activity
56	GO:0045182	18978	Consensus	0.88	Consensus	0.27	translation regulator activity
57	GO:0016491	103139	Consensus	0.86	Consensus	0.25	oxidoreductase activity
58	GO:0003774	19797	Consensus	0.83	Consensus	0.23	motor activity
59	GO:0016829	24145	Consensus	0.78	Consensus	0.23	lyase activity
60	GO:0016209	6685	Consensus	0.77	Consensus	0.21	antioxidant activity
61	GO:0009055	33126	Consensus	0.81	Consensus	0.11	electron carrier activity
62	GO:0030312	482	M1	0.69	M2	0.00	external encapsulating structure

COMBINED results sorted according to AUC

	GO term	# of cases	Best method	AUC	Best method	P20R	Explanation of GO term
1	GO:0008907	245	Consensus	0.99	M2	1.00	integrase activity
2	GO:0004871	77553	Consensus	0.92	Consensus	0.88	signal transducer activity
3	GO:0015267	7183	Consensus	0.91	Consensus	0.85	channel activity
4	GO:0004872	208752	Consensus	0.91	Consensus	0.86	receptor activity
5	GO:0051704	88280	Consensus	0.91	Consensus	0.82	multi-organism process
6	GO:0008219	98990	Consensus	0.91	Consensus	0.82	cell death
7	GO:0007154	458362	Consensus	0.90	Consensus	0.80	cell communication
8	GO:0016301	110554	Consensus	0.90	Consensus	0.83	kinase activity
9	GO:0050896	365227	Consensus	0.90	Consensus	0.80	response to stimulus
10	GO:0008150	518047	Consensus	0.90	Consensus	0.73	biological_process
11	GO:0030154	178593	Consensus	0.90	Consensus	0.78	cell differentiation
12	GO:0008152	559693	Consensus	0.89	Consensus	0.74	metabolic process
13	GO:0009056	210214	Consensus	0.89	Consensus	0.71	catabolic process
14	GO:0007275	320661	Consensus	0.89	Consensus	0.74	multicellular organismal development
15	GO:0005488	898947	Consensus	0.89	Consensus	0.66	binding
16	GO:0016020	1000479	Consensus	0.89	Consensus	0.74	membrane
17	GO:0032501	154712	Consensus	0.89	Consensus	0.81	multicellular organismal process
18	GO:0007610	33647	Consensus	0.89	Consensus	0.83	behavior
19	GO:0016874	88109	Consensus	0.89	Consensus	0.45	ligase activity
20	GO:0016740	323605	Consensus	0.89	Consensus	0.76	transferase activity
21	GO:0007124	9192	Consensus	0.89	M2	0.44	pseudohyphal growth
22	GO:0043170	487875	Consensus	0.89	Consensus	0.71	macromolecule metabolic process
23	GO:0009405	1017	Consensus	0.89	M2	1.00	pathogenesis
24	GO:0005575	727979	Consensus	0.89	Consensus	0.65	cellular_component
25	GO:0005623	202585	Consensus	0.89	Consensus	0.77	cell
26	GO:0006997	10093	Consensus	0.89	M2	0.97	nucleus organization
27	GO:0009987	649957	Consensus	0.89	Consensus	0.72	cellular process
28	GO:0009986	44472	Consensus	0.89	Consensus	0.84	cell surface
29	GO:0050789	697322	Consensus	0.89	Consensus	0.69	regulation of biological process
30	GO:0005215	130492	Consensus	0.88	Consensus	0.50	transporter activity
31	GO:0005694	120844	Consensus	0.88	Consensus	0.52	chromosome
32	GO:0030528	283322	Consensus	0.88	Consensus	0.65	transcription regulator activity
33	GO:0007047	18668	Consensus	0.88	M2	0.81	cell wall organization
34	GO:0003676	407096	Consensus	0.88	Consensus	0.56	nucleic acid binding
35	GO:0005622	394638	Consensus	0.88	Consensus	0.57	intracellular
36	GO:0006928	52075	Consensus	0.88	Consensus	0.86	cell motion
37	GO:0006810	469667	Consensus	0.88	Consensus	0.60	transport
38	GO:0005737	1249129	Consensus	0.88	Consensus	0.65	cytoplasm

39	GO:0016787	405952	Consensus	0.88	Consensus	0.62	hydrolase activity
40	GO:0005515	1135313	Consensus	0.88	Consensus	0.65	protein binding
41	GO:0005198	126605	Consensus	0.88	Consensus	0.69	structural molecule activity
42	GO:0005578	50953	Consensus	0.87	Consensus	0.73	proteinaceous extracellular matrix
43	GO:0004386	35965	Consensus	0.87	Consensus	0.46	helicase activity
44	GO:0042254	44304	Consensus	0.87	Consensus	0.88	ribosome biogenesis
45	GO:0005634	933683	Consensus	0.87	Consensus	0.56	nucleus
46	GO:0015075	85927	Consensus	0.87	Consensus	0.66	ion transmembrane transporter activity
47	GO:0030435	16477	Consensus	0.87	M2	0.41	sporulation resulting in formation of a cellular spore
48	GO:0006139	252980	Consensus	0.87	Consensus	0.50	nucleobase, nucleoside, nucleotide and nucleic acid metabolic process
49	GO:0019725	16801	Consensus	0.87	Consensus	0.20	cellular homeostasis
50	GO:0043062	15500	Consensus	0.87	Consensus	0.69	extracellular structure organization
51	GO:0003824	29099	Consensus	0.87	Consensus	0.41	catalytic activity
52	GO:0005615	108395	Consensus	0.87	M2	0.82	extracellular space
53	GO:0005624	21509	Consensus	0.87	Consensus	0.24	membrane fraction
54	GO:0030234	162899	Consensus	0.87	Consensus	0.69	enzyme regulator activity
55	GO:0000910	17082	Consensus	0.87	Consensus	0.61	cytokinesis
56	GO:0005576	285214	Consensus	0.87	Consensus	0.67	extracellular region
57	GO:0045182	25694	Consensus	0.86	Consensus	0.28	translation regulator activity
58	GO:0046903	34882	Consensus	0.86	Consensus	0.68	secretion
59	GO:0006412	42152	Consensus	0.86	Consensus	0.21	translation
60	GO:0009058	147661	Consensus	0.86	Consensus	0.67	biosynthetic process
61	GO:0007114	12912	Consensus	0.86	Consensus	0.64	cell budding
62	GO:0008565	13696	Consensus	0.85	Consensus	0.44	protein transporter activity
63	GO:0016023	15191	Consensus	0.85	Consensus	0.54	cytoplasmic membrane-bounded vesicle
64	GO:0012505	46604	Consensus	0.85	Consensus	0.89	endomembrane system
65	GO:0006944	8531	Consensus	0.85	Consensus	0.71	membrane fusion
66	GO:0003723	30748	Consensus	0.85	M2	0.50	RNA binding
67	GO:0005794	27472	Consensus	0.84	Consensus	0.56	Golgi apparatus
68	GO:0016853	29775	Consensus	0.84	Consensus	0.28	isomerase activity
69	GO:0005886	29530	Consensus	0.84	M2	0.06	plasma membrane
70	GO:0032989	22264	Consensus	0.84	Consensus	0.60	cellular component morphogenesis
71	GO:0042221	50737	Consensus	0.84	Consensus	0.23	response to chemical stimulus
72	GO:0005938	17461	Consensus	0.84	Consensus	0.47	cell cortex
73	GO:0005933	24507	Consensus	0.84	Consensus	0.46	cellular bud
74	GO:0003677	45694	Consensus	0.83	Consensus	0.23	DNA binding
75	GO:0030427	31539	Consensus	0.83	Consensus	0.40	site of polarized growth
76	GO:0004672	17648	Consensus	0.83	Consensus	0.33	protein kinase activity
77	GO:0005773	25446	Consensus	0.83	Consensus	0.22	vacuole
78	GO:0005840	36169	Consensus	0.83	Consensus	0.24	ribosome

79	GO:0016070	142192	Consensus	0.83	Consensus	0.28	RNA metabolic process
80	GO:0000746	16486	Consensus	0.83	M2	0.44	conjugation
81	GO:0016044	35037	Consensus	0.83	Consensus	0.31	membrane organization
82	GO:0003674	329842	Consensus	0.83	Consensus	0.14	molecular_function
83	GO:0005618	8689	Consensus	0.83	M2	1.00	cell wall
84	GO:0016192	48693	Consensus	0.83	Consensus	0.26	vesicle-mediated transport
85	GO:0006950	71093	Consensus	0.83	Consensus	0.23	response to stress
86	GO:0007059	21592	Consensus	0.83	Consensus	0.43	chromosome segregation
87	GO:0005739	120843	Consensus	0.83	Consensus	0.21	mitochondrion
88	GO:0051276	54607	Consensus	0.83	Consensus	0.20	chromosome organization
89	GO:0044257	28527	Consensus	0.83	Consensus	0.30	cellular protein catabolic process
90	GO:0044255	29992	Consensus	0.82	Consensus	0.19	cellular lipid metabolic process
91	GO:0006259	54363	Consensus	0.82	Consensus	0.21	DNA metabolic process
92	GO:0006519	75402	Consensus	0.82	Consensus	0.70	cellular amino acid and derivative metabolic process
93	GO:0006464	70314	Consensus	0.82	Consensus	0.23	protein modification process
94	GO:0016491	131278	Consensus	0.82	Consensus	0.12	oxidoreductase activity
95	GO:0007033	5872	Consensus	0.82	Consensus	0.29	vacuole organization
96	GO:0008233	14819	Consensus	0.82	Consensus	0.76	peptidase activity
97	GO:0070271	19752	Consensus	0.82	Consensus	0.34	protein complex biogenesis
98	GO:0007010	32379	Consensus	0.82	Consensus	0.25	cytoskeleton organization
99	GO:0003774	22196	Consensus	0.82	Consensus	0.21	motor activity
100	GO:0006350	84696	Consensus	0.81	Consensus	0.17	transcription
101	GO:0007049	72933	Consensus	0.81	Consensus	0.29	cell cycle
102	GO:0016050	9343	Consensus	0.81	Consensus	0.35	vesicle organization
103	GO:0005856	31614	Consensus	0.81	Consensus	0.27	cytoskeleton
104	GO:0006091	21768	Consensus	0.81	Consensus	0.08	generation of precursor metabolites and energy
105	GO:0016779	10915	Consensus	0.80	Consensus	0.24	nucleotidyltransferase activity
106	GO:0007165	32994	Consensus	0.80	M2	0.24	signal transduction
107	GO:0032196	6625	Consensus	0.80	Consensus	0.39	transposition
108	GO:0005730	36321	Consensus	0.80	Consensus	0.49	nucleolus
109	GO:0009055	33126	Consensus	0.80	Consensus	0.09	electron carrier activity
110	GO:0046483	19491	Consensus	0.80	M2	0.14	heterocycle metabolic process
111	GO:0006725	9209	Consensus	0.80	Consensus	0.06	cellular aromatic compound metabolic process
112	GO:0008289	10690	Consensus	0.80	M2	0.92	lipid binding
113	GO:0005783	46376	Consensus	0.79	Consensus	0.16	endoplasmic reticulum
114	GO:0007005	36372	Consensus	0.79	Consensus	0.42	mitochondrion organization
115	GO:0007126	21328	Consensus	0.79	Consensus	0.13	meiosis
116	GO:0005815	11381	Consensus	0.78	Consensus	0.17	microtubule organizing center
117	GO:0045333	11860	M1	0.78	M1	0.06	cellular respiration

118	GO:0006457	9885	Consensus	0.77	Consensus	0.23	protein folding
119	GO:0016829	34458	Consensus	0.76	Consensus	0.08	lyase activity
120	GO:0004721	6340	Consensus	0.76	M2	0.47	phosphoprotein phosphatase activity
121	GO:0044262	27231	Consensus	0.75	Consensus	0.20	cellular carbohydrate metabolic process
122	GO:0051186	17880	Consensus	0.75	M2	0.05	cofactor metabolic process
123	GO:0016209	6685	Consensus	0.75	Consensus	0.16	antioxidant activity
124	GO:0005777	8440	Consensus	0.75	M2	0.04	peroxisome
125	GO:0007031	7290	Consensus	0.72	M2	0.07	peroxisome organization
126	GO:0005740	35294	M2	0.72	Consensus	0.09	mitochondrial envelope
127	GO:0030312	482	M4	0.70	M2	0.00	external encapsulating structure
128	GO:0006766	9004	M1	0.67	Consensus	0.01	vitamin metabolic process

COMBINED results sorted according to P20R

	GO term	# of cases	Best method	AUC	Best method	P20R	Explanation of GO term
1	GO:0005618	8689	Consensus	0.83	M2	1.00	cell wall
2	GO:0009405	1017	Consensus	0.89	M2	1.00	pathogenesis
3	GO:0008907	245	Consensus	0.99	M2	1.00	integrase activity
4	GO:0006997	10093	Consensus	0.89	M2	0.97	nucleus organization
5	GO:0008289	10690	Consensus	0.80	M2	0.92	lipid binding
6	GO:0012505	46604	Consensus	0.85	Consensus	0.89	endomembrane system
7	GO:0004871	77553	Consensus	0.92	Consensus	0.88	signal transducer activity
8	GO:0042254	44304	Consensus	0.87	Consensus	0.88	ribosome biogenesis
9	GO:0006928	52075	Consensus	0.88	Consensus	0.86	cell motion
10	GO:0004872	208752	Consensus	0.91	Consensus	0.86	receptor activity
11	GO:0015267	7183	Consensus	0.91	Consensus	0.85	channel activity
12	GO:0009986	44472	Consensus	0.89	Consensus	0.84	cell surface
13	GO:0007610	33647	Consensus	0.89	Consensus	0.83	behavior
14	GO:0016301	110554	Consensus	0.90	Consensus	0.83	kinase activity
15	GO:0051704	88280	Consensus	0.91	Consensus	0.82	multi-organism process
16	GO:0005615	108395	Consensus	0.87	M2	0.82	extracellular space
17	GO:0008219	98990	Consensus	0.91	Consensus	0.82	cell death
18	GO:0007047	18668	Consensus	0.88	M2	0.81	cell wall organization
19	GO:0032501	154712	Consensus	0.89	Consensus	0.81	multicellular organismal process
20	GO:0007154	458362	Consensus	0.90	Consensus	0.80	cell communication
21	GO:0050896	365227	Consensus	0.90	Consensus	0.80	response to stimulus
22	GO:0030154	178593	Consensus	0.90	Consensus	0.78	cell differentiation
23	GO:0005623	202585	Consensus	0.89	Consensus	0.77	cell
24	GO:0016740	323605	Consensus	0.89	Consensus	0.76	transferase activity
25	GO:0008233	14819	Consensus	0.82	Consensus	0.76	peptidase activity
26	GO:0008152	559693	Consensus	0.89	Consensus	0.74	metabolic process
27	GO:0016020	1000479	Consensus	0.89	Consensus	0.74	membrane

28	GO:0007275	320661	Consensus	0.89	Consensus	0.74	multicellular organismal development
29	GO:0005578	50953	Consensus	0.87	Consensus	0.73	proteinaceous extracellular matrix
30	GO:0008150	518047	Consensus	0.90	Consensus	0.73	biological_process
31	GO:0009987	649957	Consensus	0.89	Consensus	0.72	cellular process
32	GO:0043170	487875	Consensus	0.89	Consensus	0.71	macromolecule metabolic process
33	GO:0006944	8531	Consensus	0.85	Consensus	0.71	membrane fusion
34	GO:0009056	210214	Consensus	0.89	Consensus	0.71	catabolic process
35	GO:0006519	75402	Consensus	0.82	Consensus	0.70	cellular amino acid and derivative metabolic process
36	GO:0030234	162899	Consensus	0.87	Consensus	0.69	enzyme regulator activity
37	GO:0043062	15500	Consensus	0.87	Consensus	0.69	extracellular structure organization
38	GO:0005198	126605	Consensus	0.88	Consensus	0.69	structural molecule activity
39	GO:0050789	697322	Consensus	0.89	Consensus	0.69	regulation of biological process
40	GO:0046903	34882	Consensus	0.86	Consensus	0.68	secretion
41	GO:0005576	285214	Consensus	0.87	Consensus	0.67	extracellular region
42	GO:0009058	147661	Consensus	0.86	Consensus	0.67	biosynthetic process
43	GO:0015075	85927	Consensus	0.87	Consensus	0.66	ion transmembrane transporter activity
44	GO:0005488	898947	Consensus	0.89	Consensus	0.66	binding
45	GO:0005575	727979	Consensus	0.89	Consensus	0.65	cellular_component
46	GO:0005737	1249129	Consensus	0.88	Consensus	0.65	cytoplasm
47	GO:0005515	1135313	Consensus	0.88	Consensus	0.65	protein binding
48	GO:0030528	283322	Consensus	0.88	Consensus	0.65	transcription regulator activity
49	GO:0007114	12912	Consensus	0.86	Consensus	0.64	cell budding
50	GO:0016787	405952	Consensus	0.88	Consensus	0.62	hydrolase activity
51	GO:0000910	17082	Consensus	0.87	Consensus	0.61	cytokinesis
52	GO:0006810	469667	Consensus	0.88	Consensus	0.60	transport
53	GO:0032989	22264	Consensus	0.84	Consensus	0.60	cellular component morphogenesis
54	GO:0005622	394638	Consensus	0.88	Consensus	0.57	intracellular
55	GO:0005634	933683	Consensus	0.87	Consensus	0.56	nucleus
56	GO:0005794	27472	Consensus	0.84	Consensus	0.56	Golgi apparatus
57	GO:0003676	407096	Consensus	0.88	Consensus	0.56	nucleic acid binding
58	GO:0016023	15191	Consensus	0.85	Consensus	0.54	cytoplasmic membrane-bounded vesicle
59	GO:0005694	120844	Consensus	0.88	Consensus	0.52	chromosome
60	GO:0005215	130492	Consensus	0.88	Consensus	0.50	transporter activity
61	GO:0003723	30748	Consensus	0.85	M2	0.50	RNA binding
62	GO:0006139	252980	Consensus	0.87	Consensus	0.50	nucleobase, nucleoside, nucleotide and nucleic acid metabolic process
63	GO:0005730	36321	Consensus	0.80	Consensus	0.49	nucleolus
64	GO:0004721	6340	Consensus	0.76	M2	0.47	phosphoprotein phosphatase activity
65	GO:0005938	17461	Consensus	0.84	Consensus	0.47	cell cortex
66	GO:0005933	24507	Consensus	0.84	Consensus	0.46	cellular bud
67	GO:0004386	35965	Consensus	0.87	Consensus	0.46	helicase activity

68	GO:0016874	88109	Consensus	0.89	Consensus	0.45	ligase activity
69	GO:0008565	13696	Consensus	0.85	Consensus	0.44	protein transporter activity
70	GO:0000746	16486	Consensus	0.83	M2	0.44	conjugation
71	GO:0007124	9192	Consensus	0.89	M2	0.44	pseudohyphal growth
72	GO:0007059	21592	Consensus	0.83	Consensus	0.43	chromosome segregation
73	GO:0007005	36372	Consensus	0.79	Consensus	0.42	mitochondrion organization
74	GO:0003824	29099	Consensus	0.87	Consensus	0.41	catalytic activity
75	GO:0030435	16477	Consensus	0.87	M2	0.41	sporulation resulting in formation of a cellular spore
76	GO:0030427	31539	Consensus	0.83	Consensus	0.40	site of polarized growth
77	GO:0032196	6625	Consensus	0.80	Consensus	0.39	transposition
78	GO:0016050	9343	Consensus	0.81	Consensus	0.35	vesicle organization
79	GO:0070271	19752	Consensus	0.82	Consensus	0.34	protein complex biogenesis
80	GO:0004672	17648	Consensus	0.83	Consensus	0.33	protein kinase activity
81	GO:0016044	35037	Consensus	0.83	Consensus	0.31	membrane organization
82	GO:0044257	28527	Consensus	0.83	Consensus	0.30	cellular protein catabolic process
83	GO:0007049	72933	Consensus	0.81	Consensus	0.29	cell cycle
84	GO:0007033	5872	Consensus	0.82	Consensus	0.29	vacuole organization
85	GO:0016070	142192	Consensus	0.83	Consensus	0.28	RNA metabolic process
86	GO:0016853	29775	Consensus	0.84	Consensus	0.28	isomerase activity
87	GO:0045182	25694	Consensus	0.86	Consensus	0.28	translation regulator activity
88	GO:0005856	31614	Consensus	0.81	Consensus	0.27	cytoskeleton
89	GO:0016192	48693	Consensus	0.83	Consensus	0.26	vesicle-mediated transport
90	GO:0007010	32379	Consensus	0.82	Consensus	0.25	cytoskeleton organization
91	GO:0005624	21509	Consensus	0.87	Consensus	0.24	membrane fraction
92	GO:0016779	10915	Consensus	0.80	Consensus	0.24	nucleotidyltransferase activity
93	GO:0005840	36169	Consensus	0.83	Consensus	0.24	ribosome
94	GO:0007165	32994	Consensus	0.80	M2	0.24	signal transduction
95	GO:0006950	71093	Consensus	0.83	Consensus	0.23	response to stress
96	GO:0006457	9885	Consensus	0.77	Consensus	0.23	protein folding
97	GO:0003677	45694	Consensus	0.83	Consensus	0.23	DNA binding
98	GO:0042221	50737	Consensus	0.84	Consensus	0.23	response to chemical stimulus
99	GO:0006464	70314	Consensus	0.82	Consensus	0.23	protein modification process
100	GO:0005773	25446	Consensus	0.83	Consensus	0.22	vacuole
101	GO:0006412	42152	Consensus	0.86	Consensus	0.21	translation
102	GO:0005739	120843	Consensus	0.83	Consensus	0.21	mitochondrion
103	GO:0003774	22196	Consensus	0.82	Consensus	0.21	motor activity
104	GO:0006259	54363	Consensus	0.82	Consensus	0.21	DNA metabolic process
105	GO:0051276	54607	Consensus	0.83	Consensus	0.20	chromosome organization
106	GO:0019725	16801	Consensus	0.87	Consensus	0.20	cellular homeostasis
107	GO:0044262	27231	Consensus	0.75	Consensus	0.20	cellular carbohydrate metabolic process
108	GO:0044255	29992	Consensus	0.82	Consensus	0.19	cellular lipid metabolic process

109	GO:0006350	84696	Consensus	0.81	Consensus	0.17	transcription
110	GO:0005815	11381	Consensus	0.78	Consensus	0.17	microtubule organizing center
111	GO:0005783	46376	Consensus	0.79	Consensus	0.16	endoplasmic reticulum
112	GO:0016209	6685	Consensus	0.75	Consensus	0.16	antioxidant activity
113	GO:0003674	329842	Consensus	0.83	Consensus	0.14	molecular_function
114	GO:0046483	19491	Consensus	0.80	M2	0.14	heterocycle metabolic process
115	GO:0007126	21328	Consensus	0.79	Consensus	0.13	meiosis
116	GO:0016491	131278	Consensus	0.82	Consensus	0.12	oxidoreductase activity
117	GO:0009055	33126	Consensus	0.80	Consensus	0.09	electron carrier activity
118	GO:0005740	35294	M2	0.72	Consensus	0.09	mitochondrial envelope
119	GO:0016829	34458	Consensus	0.76	Consensus	0.08	lyase activity
120	GO:0006091	21768	Consensus	0.81	Consensus	0.08	generation of precursor metabolites and energy
121	GO:0007031	7290	Consensus	0.72	M2	0.07	peroxisome organization
122	GO:0006725	9209	Consensus	0.80	Consensus	0.06	cellular aromatic compound metabolic process
123	GO:0005886	29530	Consensus	0.84	M2	0.06	plasma membrane
124	GO:0045333	11860	M1	0.78	M1	0.06	cellular respiration
125	GO:0051186	17880	Consensus	0.75	M2	0.05	cofactor metabolic process
126	GO:0005777	8440	Consensus	0.75	M2	0.04	peroxisome
127	GO:0006766	9004	M1	0.67	Consensus	0.01	vitamin metabolic process
128	GO:0030312	482	M4	0.70	M2	0.00	external encapsulating structure
