# Supplementary material for: Critical assessment of sequence-based protein-protein interaction prediction methods that do not require homologous protein sequences
Source: BMC Bioinformatics. 2009 Dec 14;10:419. doi: 10.1186/1471-2105-10-419 (PMC2803199; doi:10.1186/1471-2105-10-419)
Supplement: Additional file 3 — Detailed scheme for a two-stage 4-fold cross-validation for the consensus method. [file 1471-2105-10-419-S3.DOC]

**Two-stage cross-validation scheme for the consensus method**

Let us assume we perform a 4-fold cross-validation. Usually, one divides the data into 4 equal-sized sets and use three sets for training and one set for testing. Specifically, let us say that we are using the sets 2, 3 and 4 for training and the set 1 for testing. Then, this is done in a two-stage 4-fold cross-validation in the following way. We first aggregate the data in the sets 2, 3 and 4 and divide it into 4 equal-sized sets (say, the sets 1’, 2’, 3’ and 4’). Then we train the four methods (M1 through M4) using the data in sets 2’, 3’ and 4’ and have the trained methods predict for the set 1’. The numerical values predicted for the set 1’ by the four methods become the input vectors (i.e. vectors of 4 features) for training the consensus method. Then, the four methods are re-trained using the sets 2, 3 and 4 and used to predict for the set 1. The numerical values predicted for the set 1 by the four methods are then integrated by the above trained consensus method to generate predictions for each protein pair in the set 1. Thus, the basic idea of the consensus approach is to generate a classifier that takes into account the goodness of each prediction method in such a way that it leads to optimal combinations of their predictions. At the same time, the above training scheme for the consensus approach prevents double training to ensure unbiased prediction performance estimation.
